# Lifting the Mask: Identification of New Small Molecule Inhibitors of Uropathogenic *Escherichia coli* Group 2 Capsule Biogenesis

**DOI:** 10.1371/journal.pone.0096054

**Published:** 2014-07-01

**Authors:** Carlos C. Goller, Mehreen Arshad, James W. Noah, Subramaniam Ananthan, Carrie W. Evans, N. Miranda Nebane, Lynn Rasmussen, Melinda Sosa, Nichole A. Tower, E. Lucile White, Benjamin Neuenswander, Patrick Porubsky, Brooks E. Maki, Steven A. Rogers, Frank Schoenen, Patrick C. Seed

**Affiliations:** 1 Department. of Pediatrics, Duke University School of Medicine, Durham, North Carolina, United States of America; 2 Southern Research Specialized Biocontainment Screening Center, Southern Research Institute, Birmingham, Alabama, United States of America; 3 Specialized Chemistry Center, University of Kansas, Lawrence, Kansas, United States of America; 4 Department of Molecular Genetics and Microbiology, Duke University School of Medicine, Durham, North Carolina, United States of America; 5 Center for Microbial Pathogenesis, Duke University School of Medicine, Durham, North Carolina, United States of America; University of Helsinki, Finland

## Abstract

Uropathogenic *Escherichia coli* (UPEC) is the leading cause of community-acquired urinary tract infections (UTIs), with over 100 million UTIs occurring annually throughout the world. Increasing antimicrobial resistance among UPEC limits ambulatory care options, delays effective treatment, and may increase overall morbidity and mortality from complications such as urosepsis. The polysaccharide capsules of UPEC are an attractive target a therapeutic, based on their importance in defense against the host immune responses; however, the large number of antigenic types has limited their incorporation into vaccine development. The objective of this study was to identify small-molecule inhibitors of UPEC capsule biogenesis. A large-scale screening effort entailing 338,740 compounds was conducted in a cell-based, phenotypic screen for inhibition of capsule biogenesis in UPEC. The primary and concentration-response assays yielded 29 putative inhibitors of capsule biogenesis, of which 6 were selected for further studies. Secondary confirmatory assays identified two highly active agents, named DU003 and DU011, with 50% inhibitory concentrations of 1.0 µM and 0.69 µM, respectively. Confirmatory assays for capsular antigen and biochemical measurement of capsular sugars verified the inhibitory action of both compounds and demonstrated minimal toxicity and off-target effects. Serum sensitivity assays demonstrated that both compounds produced significant bacterial death upon exposure to active human serum. DU011 administration in mice provided near complete protection against a lethal systemic infection with the prototypic UPEC K1 isolate UTI89. This work has provided a conceptually new class of molecules to combat UPEC infection, and future studies will establish the molecular basis for their action along with efficacy in UTI and other UPEC infections.

## Introduction

Urinary tract infection (UTI) is the second leading infection in humans [1] and the most common bacterial infection in the ambulatory care setting in the United States, accounting for up to 8.6 million health care visits in 2007 [2]. Of the major causes of UTIs, *Escherichia coli* (*E. coli*) is by far the primary etiology, producing over 74.2% and 65.5% of UTIs in ambulatory and hospitalized patients, respectively [3]–[5]. Twenty-five to forty percent of first-time community-acquired UTIs are followed by recurrences caused by the same clone of UPEC [3], [6], [7]. In addition, *E. coli* also accounts for a significant proportion of sepsis and meningitis of the young and old, with the infections originating from the urinary tract or direct translocation from the gut into the bloodstream [8]. With over 100 million UTIs occurring annually throughout the world, including more than 10 million cases in U.S. adolescents and adults (per NIDDK data, [9]), UPEC accounts for substantial medical costs and morbidity worldwide.

Among all UTI cases, approximately 40-times more are treated in the outpatient setting relative to inpatient care [7]. Rising antibiotic resistance is a serious problem affecting the clinical utility of the drugs commonly available for outpatient treatment of UTIs (e.g., [10]). In the last decade, widespread use of antibiotics has resulted in an increase in resistance of *E. coli* to commonly used oral antibiotics. Whereas ampicillin and amoxicillin were once the standard of treatment in uncomplicated UTI, the rates of resistance are approaching 50% in certain parts of North America [4]. Resistance rates have also dramatically increased among UPEC against trimethoprim-sulfamethaxozole (TMP-SMX), currently the first line therapy for outpatient treatment of UTI [11], [12]. Resistance to TMP-SMX has been emerging among urinary tract isolates with rates in excess of 20% in some areas. The Infectious Diseases Society of America (IDSA) now recommends that in regions where resistance to TMP-SMX exceeds 20%, TMP-SMX should no longer be used for empirical therapy [13]. Ciprofloxacin and other fluoroquinolones are used routinely, but resistance to these agents is also on the rise (e.g., [14], [15]), and fluoroquinolone-resistant isolates of *E. coli* are often multidrug resistant [16].

Almost all UTI treated in the community occur in individuals with normal, robust immune responses to infection. Thus, a new approach to therapy may be development and institution of UTI-specific therapeutics that render microbes vulnerable to host clearance mechanisms such as the innate immunity. Multiple innate defense mechanisms are thought to participate in clearance of bacteria from the urinary tract. A robust pro-inflammatory cytokine response of IL-6 and IL-8 results from TLR4-LPS stimulation [17]–[21]. Subsequently, neutrophils are recruited into the urinary tract, producing pyuria. Complement levels increase during inflammatory conditions in the urinary tract [22] and may be an important mechanism of defense. Antimicrobial peptides (AP), including the cationic 3–5 kDa peptides called defensins, are abundant in the urine [23]. AP form pores in phospholipid bilayers but require access to the bacterial outer membrane for function [24]. Similar immune responses are activated and effective in limiting the spread of UPEC from the urinary tract to produce more disseminated disease.

The effectiveness of the innate immune response against bacteria such as *E. coli* may, however, be severely hindered by bacterial factors such as polysaccharide capsule. Capsules are well-established virulence factors for a variety of pathogens and serve to protect the cell from opsonophagocytosis and complement-mediated killing [25], [26]. In murine models, prior research has demonstrated that the K capsule of *E. coli* is a preeminent virulence determinant during UTI and bacteremia [27]–[29], as well as critical for formation of intracellular bacterial communities within the murine model of UTI. K capsules, also called K antigens, are enveloping structures composed of acidic, high-molecular-weight polysaccharides. Llobet *et al.* demonstrated that highly acidic polysaccharide capsules of *K. pneumoniae*, *P. aeruginosa*, and *S. pneumoniae* interact strongly with AP, acting as “sponges” to sequester and neutralize the AP [30]. Furthermore in murine models of UTI, we have found that K capsule contributes to multiple aspects of UTI pathogenesis, including intracellular replication [28], making inhibition of capsule biosynthesis a novel target for attenuation of UPEC virulence.

Inhibiting K capsule production may sensitize the organism to various components of the immune system. Proof-of-concept evidence comes from the demonstration that injection of purified K1 endosialiase, which enzymatically degrades the *E. coli* K1 capsule, prevented sepsis and meningitis after intraperitoneal infection of neonatal rats with *E. coli* K1 [29]. However, endosialidases may have limited therapeutic applications due to their antigenicity, poor bioavailability, and potential action on sialidated host proteins and lipids with shared linkages as the capsular sialic acids (such as those present in neural tissues, [31]). Furthermore, endosialidase has a very narrow biochemical target range, limiting its application to specific K antigen types. Chemical inhibition of K capsule production may achieve similar therapeutic results without most of these limitations.

The genomic organization of Group 2 K capsules is highly conserved with operons for assembly, export, and synthesis genes. The synthesis genes vary encoding a variety of saccharide-modifying enzymes that together change the polysaccharide composition. The assembly and export genes are conserved and encode for a multi-subunit export channel that spans the inner membrane and periplasm to direct the polysaccharide capsule through an outer membrane pore where the capsule is linked to the outer membrane through end lipidation [32]. Similarly genetic regulation of the Group 2 capsule operons is highly conserved with two promoters solely driving transcription of all of the genes needed for capsule expression [33]–[35]. Thus, small molecules inhibiting the conserved aspects of the export channel or regulation of its expression would be expected to block encapsulation of a range of serotypically diverse Group 2 encapsulated UPEC.

Here we extend our discovery process for new capsule small molecule inhibitors. We previously described a facile assay for the identification of such inhibitors that, when employed against a modest number of bioactive small molecules, revealed an inhibitor of K1 and K5 Group 2 capsule biogenesis designated as C7 [36]. We now describe the application of the assay to a large magnitude, high-throughput screen for additional broad-spectrum capsule inhibitors from which we found multiple new, chemically distinct, and highly active molecules with promising therapeutic characteristics.

## Methods

### Bacterial strains, phage, and growth conditions

All *E. coli* strains and phage used in the present study are listed in Table 1. Unless otherwise indicated, bacteria were routinely grown at 37°C in Luria-Bertani medium (LB) with shaking at 250 rpm. LB was supplemented with 1% dimethyl sulfoxide (DMSO; Acros) with or without compound. Phage lysates were prepared from 50 mL cultures of *E. coli* strains UTI89 (for K1F phage), MG1655 (for T7 phage) or DS17 (for K5 phage) and stored at 4°C over several drops of chloroform as described [37].

**Table 1:** Bacterial strains and phage used in this study.

Strain/phage	Description or relevant genotype	Reference
***Bacterial strains***		
**UTI89**	K1 *Escherichia coli* cystitis isolate	[49]
**UTI89 ΔRegion I**	Region I (*kps*) K1 capsule mutant	[28]
**UTI89 ΔRegion II**	Region II (*neu*) K1 capsule synthesis mutant	[28]
**UTI89 Δ** ***kpsM***	K1 capsule export mutant	[28]
**DS17**	*Escherichia coli* K5 pyelonephritis isolate; K5 encapsulated, susceptible to K5 specific phage	[50]
**EV36**	K-12/K1 hybrid produced by conjugation with an Hfr *kps* ^+^ strain; K1 encapsulated, susceptible to K1-specific phage.	[51]
**CFT073**	K2 *Escherichia coli* urosepsis isolate	[52]
**536**	K15 *Escherichia coli* urinary tract isolate	[53]
***Phage***		
**T7 phage (T7φ)**	Inhibited by K1 capsule	[54]
**K1F phage (K1Fφ)**	K1 capsule specific	[55]
**K5 phage (K5φ)**	K5 capsule specific	[56]

### Screen for inhibitors of bacterial capsule biogenesis

#### Primary assay

The primary assay consisted of detection of the presence and absence of the K1 capsule on the *E. coli* urinary tract isolate UTI89 under growth conditions with compounds from a large chemical library. The assay was conducted as previously described [36] with the following modifications. The primary assay was conducted in 1,536-well plate format. UTI89 Δ*kpsM*, an isogenic K1 capsule export mutant, was included as an unencapsulated control. Tetracycline, 50 µM, was used as a negative growth control. A 1∶75 dilution of overnight cultures were made in LB broth containing 0.5% DMSO, and 3 µL of this culture was added to each plate well, and plates were incubated, inverted, at 37°C for 2 hr. K1F phage stock was diluted 1∶8 in LB Broth containing 0.5% DMSO, and 1.5 µL of diluted phage (or media only) was added to the pre-plated test compound wells and appropriate control wells. The plates were centrifuged briefly, and then were incubated, inverted, at 37°C for an additional 2 hr. To increase sensitivity of detection of viable bacteria, 1 µL of a 1∶2 dilution of AlamarBlue reagent (Invitrogen, #DAL1100) in LB broth was added to each plate well. Alamar Blue, resazurin, is converted in living cells to the fluorescent molecule, resorufin. The plates were again centrifuged briefly, and then were further incubated, inverted, at 37°C for 30 min. Resorufin fluorescence was measured using excitation of 560 nm and emission of 590 nm, as per the manufacturer's recommendations.

Compounds from the NIH Molecular Libraries Small Molecule Repository (MLSMR) were utilized in the primary assay. The MLSMR collection of over 300,000 compounds generically grouped into one of the following five categories: (a) specialty sets, comprising bioactive compounds such as known drugs and toxins, (b) non-commercial compounds, mainly from academic labs, (c) targeted libraries, (d) natural products, and (e) diversity compounds [38]. The MLSMR library covers a diverse sample of the chemical space occupied by drugs and natural products, but only narrowly represents combinatorial chemical space [39]. Compounds or vehicle control (DMSO) were diluted to a final well concentration of 1∶200 in assay media. Compounds (22.5 nL in 100% DMSO) were dispensed to assay plates using an Echo non-contact dispenser (Labcyte). Compounds from the libraries were added to the plates at a final concentration of 50 µM, before the addition of bacteria or phage. Each compound was tested as a single point, and ∼1,200 compounds were tested per plate. A positive hit was defined by the compound producing greater than a 50% inhibition of K1F phage-induced lysis. Compound hits were further confirmed using an optical density-based detection method in a 96-well format as previously described [36].

#### Concentration-response to chemical inhibition

Concentration-response testing (concentration range = 0.58–300 µM) was used to confirm and characterize the primary screen hits, which was necessary to determine the number of compounds advanced to secondary screens. Compounds were plated in 1536-well microplates, and the concentration-response efficacy assay was performed as described for the primary screen, with the exception that each compound was tested in duplicate at 10 concentration points starting from 300 µM and continuing to lower concentrations by 2-fold serial dilutions. The strain UTI89 Δ*kpsM*, a K1 capsule export mutant, was evaluated with the wild-type strain as a phage-insensitive control (mimicking 100% capsule inhibition).

#### T7 phage counter assay

This secondary assay was performed as previously described [36] and was used to distinguish compounds with phage inhibitory effects from true polysaccharide capsule inhibitors. T7 phage has a nearly identical genome to K1F phage and thus a similar life cycle [40]; however, T7 phage does not encode for an endosialidase, and its entry into *E. coli* is inhibited by K capsules. Thus, the presence of a capsule inhibits T7-mediated lysis [41]. In this assay, an increase in phage-induced lysis correlates to a decrease in capsule formation. True inhibitors of capsule yielded bacteria that were susceptible to T7 phage and lysed within 2 hr of the addition of phage. However, compounds inhibiting phage replication did not promote bacterial lysis. The positive control molecule C7 (100 µM final well concentration) was used in this screen.

#### Pan Assay Interference Compound (PAINS) analysis

Groups of compound substructural features are associated with compound biological promiscuity, and compounds containing these features arise as frequent hits in biochemical high throughput screens. These molecules have been described as PAINS [42]. To determine if molecules of interest were within chemical groups with known non-specific interference with the bioassays, the structures for compounds DU001, DU003, DU005, DU007, DU008, and DU011 were retrieved from PubChem and saved in the Structure Data Format (SDF). The structures were then compared to the SYBYL PAINS compounds library, to see which, if any, of the compounds contain PAINS functional groups [43].

#### Cytotoxicity

Testing was performed essentially as previously described [36], [44]. Concentration-response testing was performed over a range 0.58–300 µM in a 386 well plate format. Bladder carcinoma 5637 cells (ATCC HTB-9) were added to the compounds, and 72 hrs later cell viability was measured using CellTiter Glo (Promega). The 50% toxic concentration (TC_50_) was determined and compared to the IC_50_ to calculate the therapeutic index. Hyamine was used as a positive cytotoxic control. All wells contained 0.5% DMSO.

#### Evaluation of off-target effects

Off target effects of lead molecules of interest were evaluated using the LeadProfilingScreen commercial assay at Eurofins Panlabs (Bothell, Washington). Reference standards were run as an integral part of each assay to ensure the validity of the results obtained. Assay results are presented as the percent inhibition of specific binding or activity (for n = 2 replicates) for the probe compound tested at a concentration of 10 µM. Details regarding the individual assays and methods are provided in File S1.

#### Orcinol assay for released capsule material

Orcinol reactivity was used as a biochemical confirmation of altered extracellular capsule after compound treatment. UTI89 or an isogenic capsule mutant was grown in 100 µM of test compound or 1% DMSO. The assay was performed as previously described [36]. The molecule C7 (100 µM final concentration) was used as a positive control. The assay was performed 3 times with replicate samples.

#### K1 antigen dot blot

K1 antigen was detected by dot blot assays of culture extracts probed with anti-K1 H46 serum. The assay was performed as previously described [36]. The experiment was repeated twice with similar results, and a representative dot blot is shown.

#### Visualization of capsule using Alcian blue staining

Overnight cultures of clinical *E. coli* strains were diluted 1∶100 in the presence of 1% DMSO or 100 µM DU011. Cultures were grown for ∼6 hrs (OD_600_ = 1.2) at 37°C. Samples were centrifuged at 13,200 RPM for 5 min. The medium was removed and the cells were resuspended in 500 µL of Tris-Acetate (pH 5) and shaken for 1 hr at 37°C. Samples were re-centrifuged, and the supernatant was concentrated ∼100 fold in Amicon 3K microconcentrators. The preparations were separated on a 7.5% SDS-PAGE gel and stained with 0.125% Alcian blue as previously described [45].

#### K5 phage assay

This assay determined if compounds found to be active in the K1F, T7, orcinol, and K1 antigen dot blot secondary assays were able to also inhibit K5 capsule biogenesis. The assay was performed in a method identical to the T7 assay test [36]. *E. coli* strain DS17, a pyelonephritis clinical isolate expressing a K5 capsule and susceptible to K5 phage (K5), was used as a K5 prototypic test strain. The degree of inhibition of phage-mediated lysis was determined based on the absorbance (OD_600_).

#### Human serum sensitization by capsule inhibitor treatment

Overnight cultures of UTI89 were diluted 1∶100 and grown with or without 50 µM compound for approximately 1.5 hrs on a shaker at 37°C. Then 25 µL of anonymous, non-identified, sterile filtered pooled human serum (purchased from Equitech Bio) was added per 100 µL of growth media. This was returned to the shaker for another 3 hrs, after which 20 µL of 5 mg/mL MTT (3-(4,5-Dimethylthiazol-2-yl)-2,5- diphenyltetrazolium bromide) was added. MTT is reduced to purple formazan by bacterial reductase enzymes, thus measuring viable bacteria. This was shaken for another 15 min at 37°C. The sample was spun to remove the growth media, followed by two washes with PBS. The formazan crystals were dissolved in 100 µL of DMSO and measured at OD_570_.

#### Murine UPEC sepsis model and treatment with DU011

Groups of five 6–7 week old C57BL/6NCr female mice (purchased from Frederick National Laboratory for Cancer Research) were injected subcutaneously twice daily with 100 µL of 1% DMSO (vehicle control) or DU011 (1 mg/ml) starting 12 hrs prior to the intra peritoneal infection. Weights were recorded twice daily to monitor health of the animals and tolerance to the compound. Mice were challenged by intraperitoneal injection with 10^8^ CFU of the indicated *E. coli* UTI89 in 100 µL of PBS. Briefly, cultures were prepared by diluting overnight cultures (18 hrs) 1∶100 into 3 mL of LB supplemented with 1% DMSO final or 100 µM DU011 (1% DMSO final). Shaken cultures were grown at 37°C for 6 hours to an OD_600_ of 1.2, and then cells were pelleted and resuspended in 1 mL of PBS. Absorbance was adjusted to OD_600_ of 0.8 in PBS, and the cultures were then diluted 1∶10 in sterile PBS. Animals were also given an intraperitoneal dose of 1% DMSO or DU011, in a site different than the administration of bacteria, to ensure sufficient systemic delivery of drug. Animal survival was assessed after 12 hours. Surviving mice were re-dosed according to the treatment groups and continued to be monitored. The experiment was concluded 48 hours post infection. The entire experiment was repeated with similar results.

Throughout each experiment, animals were monitored each 6 hours from the time of infection until conclusion for serious morbidity, including ruffled fur, decreased activity, slowed respirations, and ill appearance. Animals were provided gel packs for easily accessible additional hydration throughout the experiments. When a moribund state was suspected or anticipated by these criteria, animals were immediately euthanized to minimize potential pain and/or suffering. Of the total animals with an outcome categorized as death, the following numbers were euthanized for terminal morbidity prior to septic death (euthanized/total deaths): 3/10, no prior treatment; 3/5 prior chemical treatment of bacteria alone; 0/2 prior chemical treatment of mice alone. Euthanasia for all animals was through complete respiratory cessation using the inhaled anesthetic isoflurane followed by secondary assurance of death using bilateral thoracotomy. All animal experiments were conducted with prior approval from the Institutional Animal Care and Use Committee of Duke University.

### Statistical analyses

Results were calculated as averages and standard deviations of the means using the Graph Pad Prism 5 software package (San Diego, CA). Nonparametric t-tests were used for statistical analysis of data and calculation of *p*-values using Graph Pad Prism 5 or Graph Pad online calculators. Significant differences are highlighted with a single asterisk when the p value is less than 0.05, with two asterisks when the p value is less than 0.01, and three asterisks when the p value is less than 0.001.

## Results

### Primary screen for novel capsule inhibitors

Our initial screen of 2,195 compounds from the Developmental Therapeutics Program at the National Cancer Institute successfully identified a small-molecule inhibitor of uropathogenic *E. coli* Group 2 capsule biogenesis. We described this compound, termed C7, in a previous report as proof-of-principle that small-molecule inhibition of capsule biogenesis is possible and that these novel anti-infectives can block encapsulation and attenuate a pathogen through exposure to host innate immune factors [36]. Based on this proof-of-concept, the primary assay was adapted to a 1,536-well format with the modifications for high-throughput screening necessary to search for additional active molecules in significantly larger chemical repositories. The K1 encapsulated strain of uropathogenic *E. coli* UTI89 was grown in a 1,536-well plate format in the presence of 50 µM compounds. After an initial growth step, K1F phage specific for K1 capsule was added. Compounds with no effect on capsule biogenesis allowed the growth of organisms with an intact capsule that were subsequently lysed by the addition of the K1F phage. However, those compounds that presumably inhibited capsule biogenesis and allowed growth of the unencapsulated organism did not lyse with the addition of the K1F phage. These compounds were then selected for a secondary assay.

**Figure 1:**
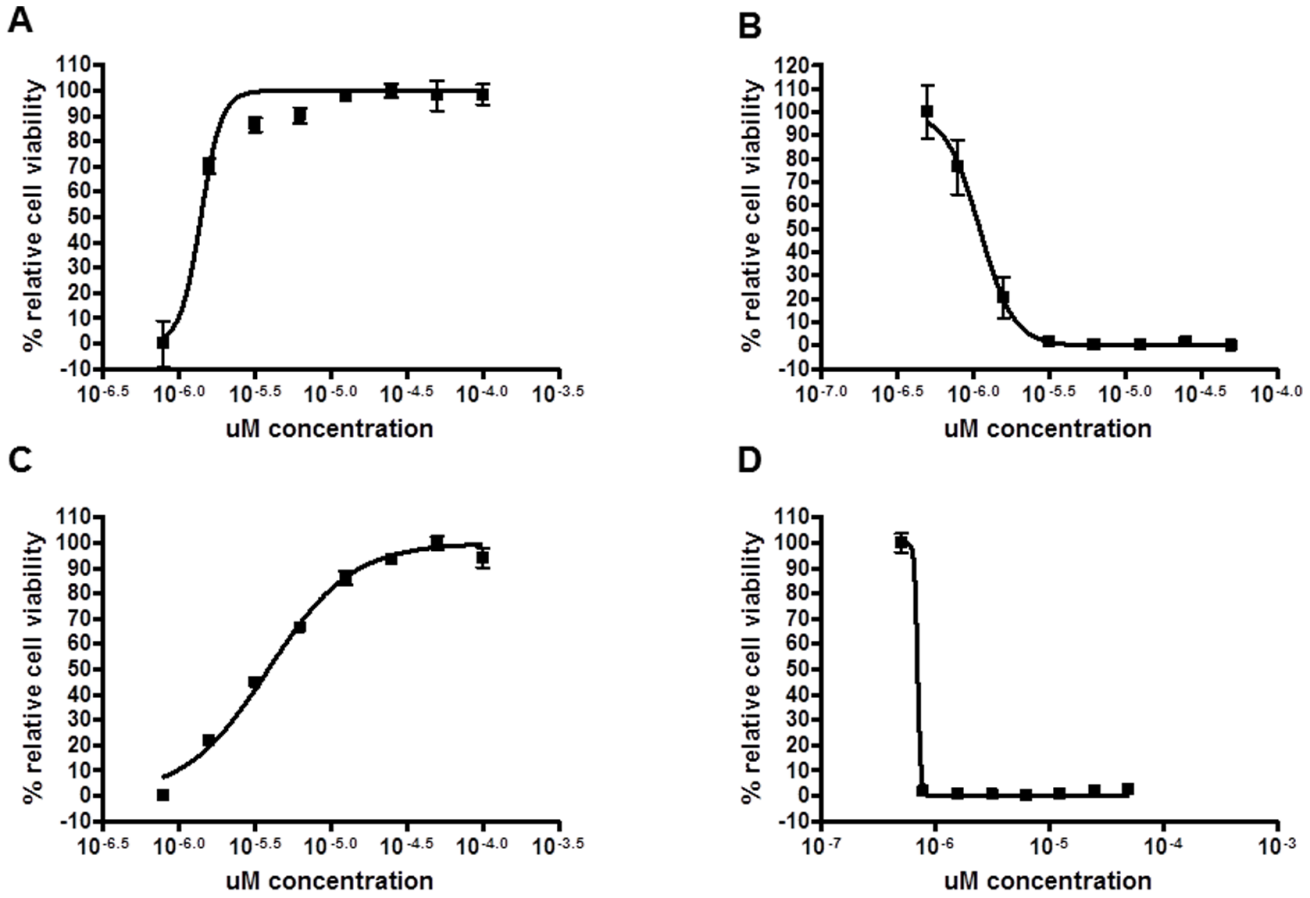
Concentration-response inhibition of K1 and T7 phage-mediated cell lysis. K1 (**A, C**) and T7 (**B, D**) phage activity in *E. coli* strain UTI89 or EV36 (K1∶K-12 hybrid strain), respectively, following treatment with various concentrations of DU003 (**A, B**) or DU011 (**C, D**).

In total, 338,740 compounds were screened in the primary assay (using K1F phage), and 1,767 compounds associated with resistance to phage lysis (0.52% of total) were tested in concentration-response format. Of these, 29 compounds passed concentration-response validation (1.6% of compounds passing the primary screen), and 6 were selected after demonstrating high activity in the T7 phage secondary assay, the reciprocal phage assay in which chemical unencapsulation sensitizes a K1∶K12 hybrid strain, EV36, to lysis by the T7 phage. Concentration response curves for 2 of these compounds are shown in Figure 1. Compounds with promising chemical structures and low cytotoxicity to cultured bladder epithelial cells were selected for further characterization. As seen in Table 2, the activity of these compound hits in primary and secondary assays is consistent with molecules with capsule inhibitory action. Cytotoxicity data further indicate that these molecules are non-toxic to cultured bladder epithelial cells. Furthermore, these new compounds, designated DU001, DU003, DU005, DU007, DU008, and DU011, are more active in the phage assays than the original proof-of-concept agent C7, with IC_50_ values in the micromolar range.

**Table 2:** Structure, K1 and T7 phage activity, cytotoxicity, and selectivity of capsule inhibitor compounds.

Compound	CID	IC50 K1 phage (in µM)	IC50 T7 phage (in µM)	Cytotoxicity (CC50 in µM)	Selectivity (CC50/IC50)
DU001	1247114	10.6±2.0	0.26±0.18	>100	>9.4
DU003	18109210	1.38±0.02	1.0±0.03	239±89	183
DU005	24235566	26.1±1.57	1.57±0.01	>100	>3.8
DU007	46254879	22.6±5.4	2.5±0.02	>100	4.4
DU008	4483668	3.3±0.76	0.54±0.7	45.08	13.7
DU011	23602075	4.5±2.5	0.69±0.78	51.6±13.6	13.3

Values shown correspond to the average of at least 3 replicates ± SEM. CID indicates the PubChem Compound Identification.

### Identification of potential Pan Assay Interference Compounds (PAINS) among capsule inhibitor hits

A number of compound substructural features have been identified that are associated with compound biological promiscuity, and, in particular, compounds with certain structural features appear as frequent hits in biochemical high throughput screens. These molecules have been described as PAINS {Merging Citations}. Although PAINS may remain useful hits, we sought to prioritize the compound hits DU001, DU003, DU005, DU007, DU008, and DU011 by identifying and removing PAINS-like molecules from our prioritization. Three of the six most active and selective compound hits, DU005, DU007, and DU008, are considered PAINS [40], and thus were not submitted for additional biological characterization.

### Confirmation of primary hits and spectrum of activity

**Figure 2:**
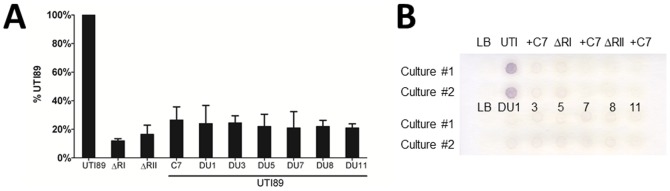
Biochemical and immunologic verification of *E. coli* Group 2 capsule inhibition through small molecules. **A**) Orcinol reactivity of capsular material released by mild acid treatment of cultures grown with 1% DMSO vehicle (UTI89 and genetic capsule mutants) or 100 µM C7, DU001, DU003, DU005, DU007, DU008, or DU011. Data represent independent experiments performed in duplicate. Treatment of K1 strain UTI89 with compounds reduces amount of orcinol-reactive polysaccharides on surface of bacteria by ∼80%. **B**) Whole-cell anti-K1 dot blots of cultures of UTI89 or indicated genetic capsule mutants treated with 1% DMSO or 100 µM DU001, DU003, DU005, DU007, DU008, or DU011 indicate that treatment of cultures with compounds reduces K1 reactive material to levels comparable to those of genetic capsule mutants. ΔRI and ΔRII indicate a complete deletion of Region I of the capsule *kps* and Region II capsule *neu* loci, respectively.

Compounds were further characterized by determining biochemically the level of surface capsule upon compound treatment of a wild-type K1 encapsulated UPEC strain. We used mild-acid to release capsule from cultures of UTI89 grown in the presence of 100 µM compound. We then used the orcinol reagent to quantify the amount of released material. As shown in Figure 2A, orcinol reactivity as % of wild-type for compound treatment indicates that cultures treated with capsule inhibitors had decreased surface reactive material, similar to levels observed for capsule synthesis and assembly mutants.

As another independent measure of capsule inhibition by the DU compounds, K1 antigen was evaluated using whole-cell dot blots with anti-K1 serum. In all cases, treatment with these molecules reduced reactivity to levels resembling those of genetic capsule mutants (Figure 2B). The combined results from the phage, orcinol, and immune-dot blots demonstrate that these molecules inhibit normal capsule production and assembly, significantly reducing the amount of surface assembled capsule.

**Figure 3:**
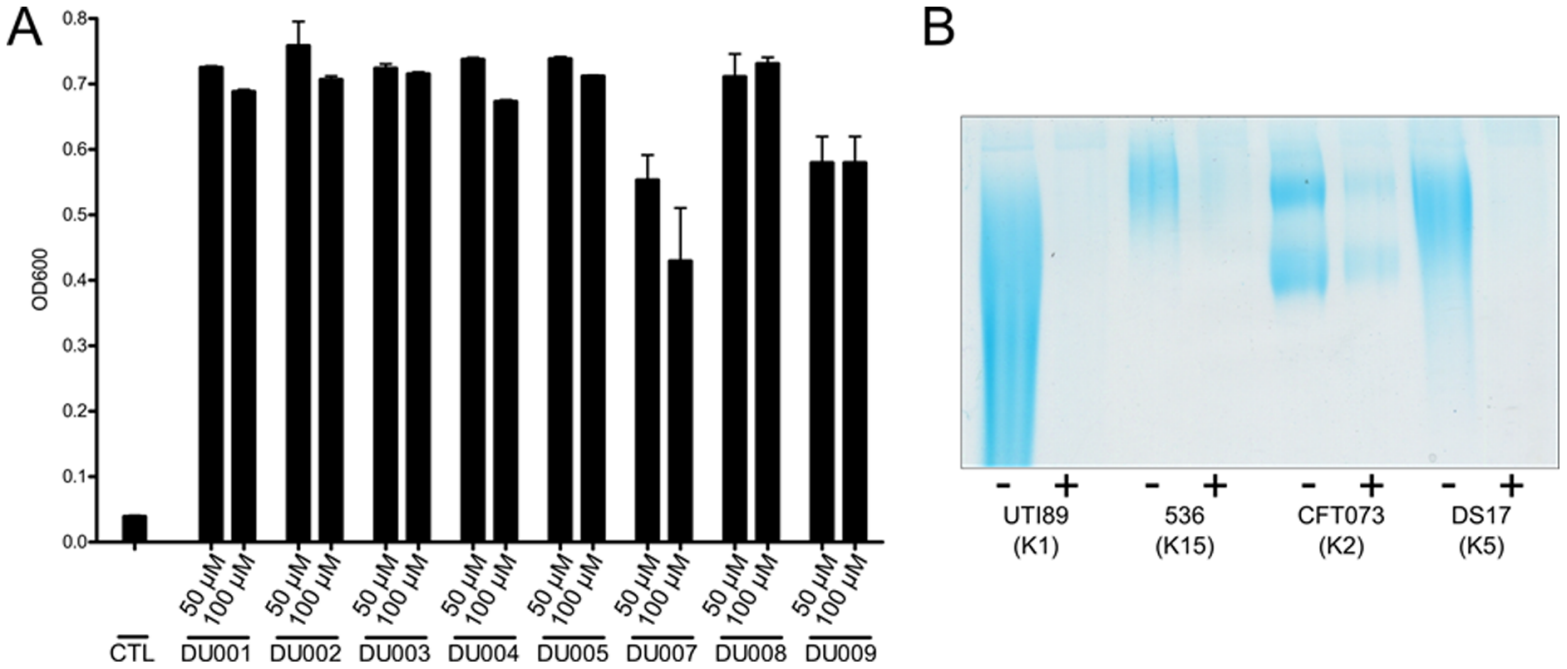
Inhibitor treatment decreases capsule production in different pathogenic *E. coli* serotypes. **A**) K5 *E. coli* was grown in vehicle or with different inhibitors (50 and 100 µM) and then challenged with K5 lytic phage, which results in cell death in the presence of capsule. Growth was measured by absorbance at OD_600_. **B**) Capsular material was isolated from multiple strains grown with and without inhibitor DU011 (200 µM). Capsule preparations were performed in at least 3 independent trials. A single representative image is shown.

A major consideration was whether the inhibitors of K1 encapsulation were also able to inhibit the production of other important *E. coli* Group 2 capsule types. To confirm the range of activity, we first demonstrated that treatment with each of confirmed inhibitory molecules also inhibited K5 phage infection of a K5 capsule-expressing strain, DS17 (Figure 3A). We next determined if leading capsule inhibitors would also inhibit the production of capsule in non-K1 serotypes. Capsular material was isolated from strains 536 (K15 serotype), CFT073 (K2 serotype) and DS17 (K5 serotype), and UTI89 (K1 serotype) in the presence and absence of capsule inhibitors. Capsule was isolated from the same amount of bacteria after growth in vehicle or with inhibitor DU011 (200 µM). Isolated material was separated on a polyacrylamide gel and stained with the cationic dye alcian blue. As shown in Figure 3B, capsule was reduced in each strain following growth with DU011, regardless of serotype. Although visibly reduced in capsule after growth in DU011, the reduction in capsular material from treated CFT073 appeared slightly less than the other serotypes tested. A similar reduction in extracted capsule was also seen with DU003 (data not shown).

### Chemical inhibition sensitized UPEC to serum-mediated killing

**Figure 4:**
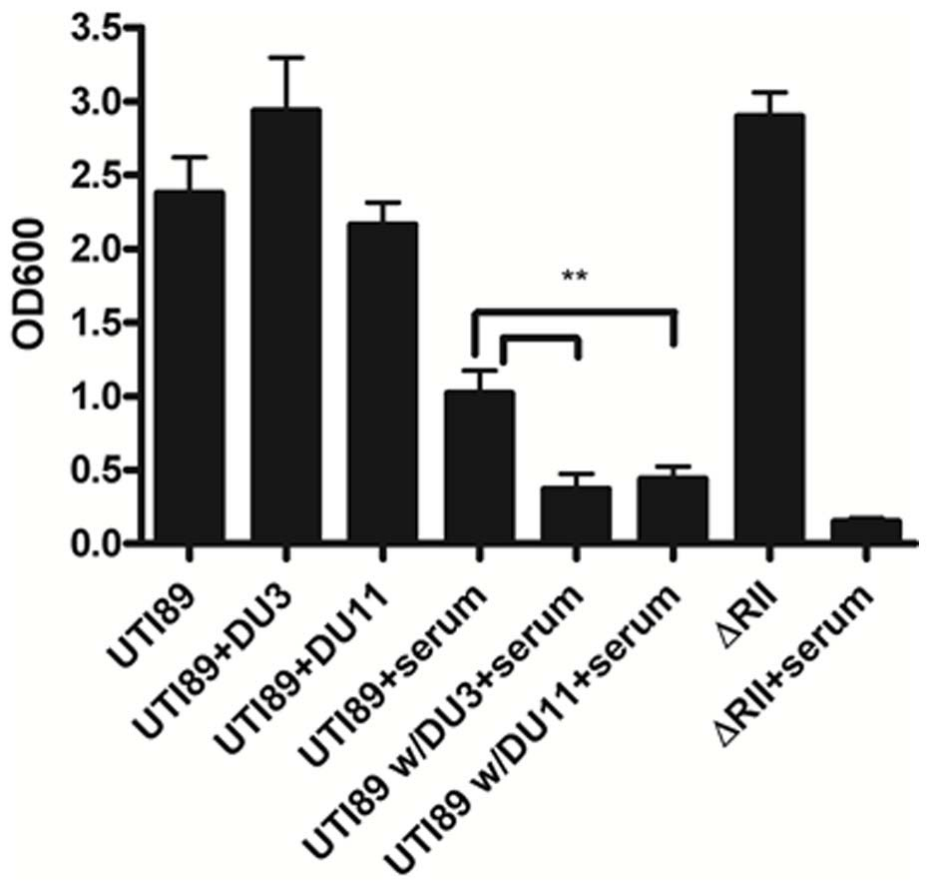
Capsule inhibitors sensitize UPEC K1 strain to serum-mediated killing. *E. coli* UTI89 and genetic capsule mutants were grown in the presence and absence of DU003 or DU011 at 50 µM and exposed to human serum. Bacterial metabolism and viability was measured using MTT (3-(4,5-Dimethylthiazol-2-yl)-2,5- diphenyltetrazolium bromide). UTI89 grown in the presence of 50 µM DU003 or DU011 were significantly more sensitive to pooled human serum compared to control UTI89 (** p = 0.0067). This was similar to the serum sensitivity of the capsule mutant UTI89 ΔRII. ΔRI and ΔRII indicate a complete deletion of Region I of the capsule *kps* and Region II capsule *neu* loci, respectively.

Capsule offers protection against serum-mediated killing. In order to test whether compound treatment of UPEC increased serum-mediated killing, an *in vitro* serum resistance assay was used. As shown in Figure 4, pooled human serum decreased the amount of viable UPEC as measured by an MTT metabolic activity assay. Treatment of UPEC with either 50 µM DU003 or DU011 further increased serum-mediated killing, thus significantly reducing the measurable bacteria as compared to DMSO treated cells (p = 0.0067). This decrease was similar to the serum sensitivity observed for a genetic capsule mutant (Δ*neu*).

### DU003 & DU011 are biologically selective

DU003 and DU011 were submitted to the Eurofins Panlabs LeadProfilingScreen to assess off-target pharmacology [42]. Both compounds were tested in duplicate at 10 µM concentration and showed no significant activity across the panel of 68 targets (i.e., <32% inhibition for DU011 and <30% inhibition for DU003, except for one target, the norepinephrine transporter, for which 58% inhibition was observed; full data and assays are provided in File S1). In review of PubChem data, DU011 (CID23602075) is reported to have shown activity in only 21 of 467 (4.5%, as of September 5, 2013) bioassays in which it was tested (assays unrelated to the current project). Per PubChem, DU003 (CID18109210) is reported to have shown activity in only 5 of 427 (1.2%, as of September 5, 2013) bioassays in which it was tested (assays unrelated to the current project). Together, these results suggest that DU011 and DU003 are not biologically promiscuous compounds.

### Attenuation of *E. coli* disseminated infection by DU011

**Figure 5:**
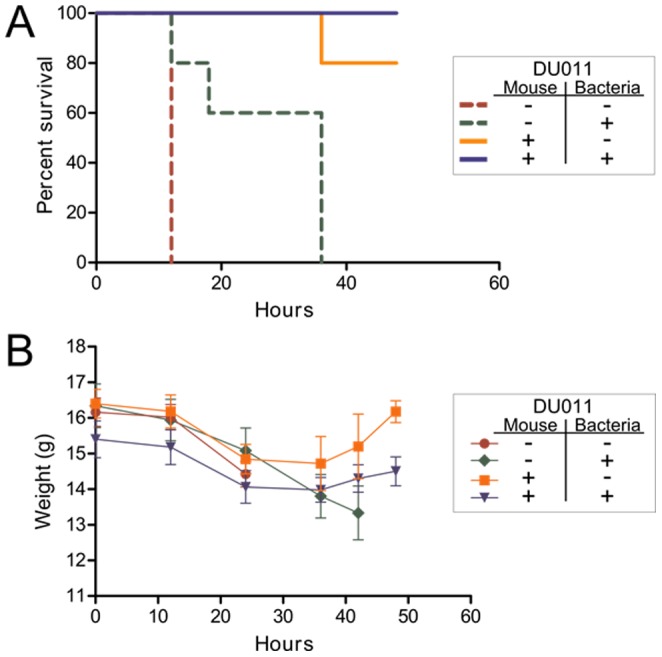
DU011 protects mice against a lethal dose of K1 *E. coli*. **A**) C57BL/6 mice were administered subcutaneous 1% DMSO (control) or DU011 (100 µL of 1 mg/ml in 1% DMSO) 12 hours prior to lethal intraperitoneal injection with 10^8^ CFU of UTI89 prepared in media containing 1% DMSO or DU011 (200 µM in 1% DMSO). Surviving animals continued to receive DMSO or DU011 each 12 hours through the course of the experiment, according to their groups (**B**) Weight was monitored during DMSO and DU011 administration and after infection.

In order to test the ability of these compounds to prevent lethal systemic *E. coli* infection in mice, we selected DU011 for animal testing based on its favorable solubility, permeability, and plasma and microsome stability profiles (Table 3). As shown in Figure 5A, C57BL/6 mice were administered DU011 or 1% DMSO 12 hours prior to lethal challenge with 10^8^ CFU of UPEC UTI89 by intraperitoneal injection. All previously untreated mice receiving non-pretreated bacteria were moribund or died within 24 hr post-infection. In contrast, pretreatment of the bacteria and mice with DU011 conferred complete protection with 100% survival for the duration of the experiments (48 hrs) and recovery of pre-infection weights (Figure 5B), similar to the 100% survival of animals administered UTI89 ΔRII, an unencapsulated isogenic mutant (data not shown). Administration of subcutaneous DU011 and untreated UTI89 provided 80% survival with stabilization of weights at the end of the experiment. Pre-treatment of bacteria without pretreatment of the mice produced 50% survival with ongoing weight loss among surviving animals. Mice tolerated DU011 without evident side effects. These data indicate that pretreatment of mice with DU011 was able to significantly reduce mortality due to disseminated *E. coli* infection, demonstrating the potency of polysaccharide inhibition *in vivo*.

**Table 3:** Solubility, permeability and plasma and microsome stability.

Compound	PubChem CID	Aqueous Solubility^1^ (µg/mL)	Hepatic Microsome Stability^2^ (Human/Mouse)	Aqueous Solubility^3^ (µg/mL)	PAMPA Permeability Pe^4^ (×10−6 cm/s) (Donor pH: 5.0/6.2/7.4)	Plasma Stability^5^ (Human/Mouse)
**DU001**	1247114	<0.01	38.73/0.56	<0.01	749/1303/566	90.09/92.69
**DU003**	18109210	104.1	57.86/14.57	>126	35/183/230	100/86.86
**DU011**	23602075	92.6	89.64/74.32	68	14/209/52	95.31/78.39

1 Measured in 1× PBS.

2 Percent remaining at 1 hr.

3 Measured in LB medium.

4 Acceptor pH: 7.4.

5 Percent remaining at 3 hrs.

## Discussion

*E. coli* infections play a significant role in community-acquired UTI with substantial morbidity and associated costs. With a diminishing arsenal of antibiotics available for the treatment of UTI, new therapeutics are in great demand. Anti-virulence agents capable of specifically attenuating a pathogenic organism during its infectious cycle hold great potential as they may spare the microbiota in commensal niches.

Previous work in our lab and in others has highlighted the importance of capsular polysaccharides in the pathogenesis of uropathogenic *E. coli* [27]–[29]. Group 2 and 3 capsules are highly conserved and represent the predominant circulating capsule types [46]. We have previously described the identification and characterization of a novel agent designated C7 that is active (IC_50_ between 12.5–25 µM), blocks the production of K1 and K5 capsule biogenesis, and lacks obvious toxicity to cultured bladder epithelial cells [36]. We have since conducted a high-throughput screen for additional broad-spectrum capsule inhibitors, finding several structurally distinct and highly active new molecules with promising therapeutic characteristics.

Herein, we described the initial identification and characterization of these new small molecules. This group of capsule inhibitors features lower IC_50_ values and improved solubility, permeability, and plasma and microsome stability profiles (Table 3; File S2). We have demonstrated their activity in assays with human serum (Figure 4). Most importantly, dosing of mice with DU011 had no detectable adverse effects on the animals and protected against a lethal *E. coli* systemic challenge (Figure 5). This new approach may provide the basis for the next pre-clinical steps to test these inhibitors as UTI-specific therapeutics that render microbes vulnerable to host clearance mechanisms such as innate immunity, enhancing the adaptive immune responses in the process by allowing greater engagement of the immune system. While dosing and delivery will need to be evaluated in future pre-clinical pharmacokinetic and pharmacodynamics studies, we believe these data highlight the potential use of capsule inhibitors as specific anti-virulence therapeutics. The compounds listed in Tables 2 and 3 are synthetically amenable lead compounds and work is currently underway to improve upon their properties. Synthesis of large lots of DU011 and other compound hits has made animal testing possible as well as facilitated work on the mechanism of action of these compounds. This will aid in the identification of their biological target and will advance our understanding of capsule biogenesis and regulation in *E. coli* and other organisms with similarly conserved capsule loci. This could lead to the attenuation of diverse encapsulated organisms sharing similar capsule assembly and regulatory mechanisms. The generation of novel highly active small-molecule inhibitors of capsule biogenesis will also aid in our understanding of the role of the innate and adaptive immune systems in control of encapsulated bacterial pathogens during systemic infections. A better understanding of how DU003, DU011 and others affect the interaction of the bacterium with host immune responses will significantly aid in the development better anti-infectives that not only attenuate the organism, but also actively engage the host immune system to promote clearance.

## Supporting Information

File S1 **Ricera LeadProfiling Screen Data Tables.** Data for screens of off target effects by DU003 and DU011 are provided.
(PDF)

File S2 **Experimental procedures for aqueous and LB medium solubility, hepatic microsome stability, PAMPA permeability, and plasma stability.** Additional methodological details are provided for these assays.
(PDF)
