# Supplementary material for: Lifting the Mask: Identification of New Small Molecule Inhibitors of Uropathogenic Escherichia coli Group 2 Capsule Biogenesis
Source: PLoS One. 2014 Jul 1;9(7):e96054. doi: 10.1371/journal.pone.0096054 (PMC4077706; doi:10.1371/journal.pone.0096054)
Supplement: File S1 — Ricera LeadProfiling Screen Data Tables. Data for screens of off target effects by DU003 and DU011 are provided. (PDF) [file pone.0096054.s001.pdf]

SI1-Supporting Information - Goller *et al.*

Ricera LeadProfilingScreen

Assays were performed at Eurofins Panlabs to identify potential eukaryotic, off-target effects of capsule inhibitors of interest. An overview of this commercial service can be found at: https://www.eurofinspanlabs.com/Catalog/Products/ProductDetails.aspx?prodId=0aCrd3Mu4RA%3D

The assays are based on radioligand binding, employing the compounds DU003 and DU011.

Methods employed in this study have been adapted from the scientific literature to maximize reliability and reproducibility. As part of the assays, reference standards were run for each assay to ensure the validity of the results obtained.

Data are presented as the percent inhibition of control radioligands.

DU003 Significant Inhibition

No significant inhibition was detected.

DU011 Significant Inhibition:

Assay Name	Species	Concentration	%Inhibition
Transporter, Norepinephrine	Human	10 μM	58

Experimental data are provided in the next sections.

A complete list of the radioligand assays and assay methods are provided in the section following additional experimental data.

Experimental Results-DU003

Cat #	Assay Name	Batch*	Spec.	Rep.	Conc.	% Inh.	IC_{50}*	K_i	n_H	R
**Compound: DU003**										
200510	Adenosine A_1	314096	hum	2	10 µM	28				
200610	Adenosine A_{2A}	314151	hum	2	10 µM	19				
200720	Adenosine A_3	314213	hum	2	10 µM	29				
203100	Adrenergic α_{1A}	314153	rat	2	10 µM	16				
203200	Adrenergic α_{1B}	314154	rat	2	10 µM	0				
203400	Adrenergic α_{1D}	314156	hum	2	10 µM	2				
203620	Adrenergic α_{2A}	314100	hum	2	10 µM	-1				
204010	Adrenergic β_1	314161	hum	2	10 µM	2				
204110	Adrenergic β_2	314163	hum	2	10 µM	3				
285010	Androgen (Testosterone) AR	314097	rat	2	10 µM	-5				
212510	Bradykinin B_1	314221	hum	2	10 µM	3				
212620	Bradykinin B_2	314223	hum	2	10 µM	6				
214510	Calcium Channel L-Type, Benzothiazepine	314226	rat	2	10 µM	2				
214600	Calcium Channel L-Type, Dihydropyridine	314169	rat	2	10 µM	32				
216000	Calcium Channel N-Type	314227	rat	2	10 µM	-16				
217030	Cannabinoid CB_1	314171	hum	2	10 µM	13				
219500	Dopamine D_1	314173	hum	2	10 µM	5				
219700	Dopamine D_{2S}	314174	hum	2	10 µM	4				
219800	Dopamine D_3	314297	hum	2	10 µM	4				
219900	Dopamine $D_{4.2}$	314238	hum	2	10 µM	7				
224010	Endothelin ET_A	314240	hum	2	10 µM	8				
224110	Endothelin ET_B	314241	hum	2	10 µM	3				
225510	Epidermal Growth Factor (EGF)	314243	hum	2	10 µM	6				
226010	Estrogen ERα	314245	hum	2	10 µM	9				
226600	$GABA_A$, Flunitrazepam, Central	314115	rat	2	10 µM	16				
226500	$GABA_A$, Muscimol, Central	314175	rat	2	10 µM	9				
228610	$GABA_{B1A}$	314248	hum	2	10 µM	5				
232030	Glucocorticoid	314321	hum	2	10 µM	-2				
232700	Glutamate, Kainate	314298	rat	2	10 µM	11				
232810	Glutamate, NMDA, Agonism	314250	rat	2	10 µM	9				
232910	Glutamate, NMDA, Glycine	314253	rat	2	10 µM	-1				
233000	Glutamate, NMDA, Phencyclidine	314178	rat	2	10 µM	6				
239610	Histamine H_1	314180	hum	2	10 µM	2				

Note: Items meeting criteria for significance (≥50% stimulation or inhibition) are highlighted.
* Batch: Represents compounds tested concurrently in the same assay(s).
R=See Remarks (if any) at end of this section.
ham=Hamster; hum=Human

Experimental Results-DU003

Cat #	Assay Name	Batch*	Spec.	Rep.	Conc.	% Inh.	IC_{50}*	K_i	n_H	R
239710	Histamine H_2	314258	hum	2	10 µM	2				
239820	Histamine H_3	314302	hum	2	10 µM	14				
241000	Imidazoline I_2, Central	314181	rat	2	10 µM	-1				
243520	Interleukin IL-1	314186	mouse	2	10 µM	3				
250460	Leukotriene, Cysteinyl $CysLT_1$	314262	hum	2	10 µM	0				
251600	Melatonin MT_1	314266	hum	2	10 µM	3				
252610	Muscarinic M_1	314320	hum	2	10 µM	1				
252710	Muscarinic M_2	314183	hum	2	10 µM	-8				
252810	Muscarinic M_3	314185	hum	2	10 µM	14				
257010	Neuropeptide Y Y_1	314271	hum	2	10 µM	5				
257110	Neuropeptide Y Y_2	314303	hum	2	10 µM	-3				
258590	Nicotinic Acetylcholine	314188	hum	2	10 µM	13				
258700	Nicotinic Acetylcholine α1, Bungarotoxin	314189	hum	2	10 µM	8				
260130	Opiate δ_1 (OP1, DOP)	314191	hum	2	10 µM	0				
260210	Opiate κ(OP2, KOP)	314304	hum	2	10 µM	14				
260410	Opiate µ(OP3, MOP)	314194	hum	2	10 µM	3				
264500	Phorbol Ester	314195	mouse	2	10 µM	-1				
265010	Platelet Activating Factor (PAF)	314197	hum	2	10 µM	4				
265600	Potassium Channel [K_{ATP}]	314201	ham	2	10 µM	9				
265900	Potassium Channel hERG	314202	hum	2	10 µM	-11				
268420	Prostanoid EP_4	314208	hum	2	10 µM	-4				
268700	Purinergic P_{2X}	314300	rabbit	2	10 µM	-2				
268810	Purinergic P_{2Y}	314301	rat	2	10 µM	8				
270000	Rolipram	314203	rat	2	10 µM	9				
271110	Serotonin (5-Hydroxytryptamine) $5\text{-}HT_{1A}$	314275	hum	2	10 µM	4				
271700	Serotonin (5-Hydroxytryptamine) $5\text{-}HT_{2B}$	314205	hum	2	10 µM	11				
271910	Serotonin (5-Hydroxytryptamine) $5\text{-}HT_3$	314281	hum	2	10 µM	-11				
278110	Sigma σ_1	314095	hum	2	10 µM	5				
255520	Tachykinin NK_1	314269	hum	2	10 µM	7				
285900	Thyroid Hormone	314309	rat	2	10 µM	-3				
220320	Transporter, Dopamine (DAT)	314094	hum	2	10 µM	8				
226400	Transporter, GABA	314450	rat	2	10 µM	10				
204410	Transporter, Norepinephrine (NET)	314086	hum	2	10 µM	13				
274030	Transporter, Serotonin (5-Hydroxytryptamine) (SERT)	314284	hum	2	10 µM	8				

Note: Items meeting criteria for significance (≥50% stimulation or inhibition) are highlighted.
* Batch: Represents compounds tested concurrently in the same assay(s).
R=See Remarks (if any) at end of this section.
ham=Hamster; hum=Human

Experimental Results-DU011

Cat #	Assay Name	Batch*	Spec.	Rep.	Conc.	% Inh.	IC_{50}*	K_i	n_H	R
**Compound: DU011**										
200510	Adenosine A_1	314096	hum	2	10 µM	-17				
200610	Adenosine A_{2A}	314151	hum	2	10 µM	-6				
200720	Adenosine A_3	314213	hum	2	10 µM	-5				
203100	Adrenergic α_{1A}	314153	rat	2	10 µM	-3				
203200	Adrenergic α_{1B}	314154	rat	2	10 µM	14				
203400	Adrenergic α_{1D}	314156	hum	2	10 µM	7				
203620	Adrenergic α_{2A}	314100	hum	2	10 µM	-14				
204010	Adrenergic β_1	314161	hum	2	10 µM	3				
204110	Adrenergic β_2	314163	hum	2	10 µM	8				
285010	Androgen (Testosterone) AR	314097	rat	2	10 µM	5				
212510	Bradykinin B_1	314221	hum	2	10 µM	12				
212620	Bradykinin B_2	314223	hum	2	10 µM	4				
214510	Calcium Channel L-Type, Benzothiazepine	314226	rat	2	10 µM	-1				
214600	Calcium Channel L-Type, Dihydropyridine	314169	rat	2	10 µM	18				
216000	Calcium Channel N-Type	314227	rat	2	10 µM	-18				
217030	Cannabinoid CB_1	314171	hum	2	10 µM	20				
219500	Dopamine D_1	314173	hum	2	10 µM	4				
219700	Dopamine D_{2S}	314174	hum	2	10 µM	-6				
219800	Dopamine D_3	314297	hum	2	10 µM	6				
219900	Dopamine $D_{4.2}$	314238	hum	2	10 µM	0				
224010	Endothelin ET_A	314240	hum	2	10 µM	-4				
224110	Endothelin ET_B	314241	hum	2	10 µM	3				
225510	Epidermal Growth Factor (EGF)	314243	hum	2	10 µM	8				
226010	Estrogen ERα	314245	hum	2	10 µM	13				
226600	$GABA_A$, Flunitrazepam, Central	314115	rat	2	10 µM	12				
226500	$GABA_A$, Muscimol, Central	314175	rat	2	10 µM	4				
228610	$GABA_{B1A}$	314248	hum	2	10 µM	7				
232030	Glucocorticoid	314321	hum	2	10 µM	16				
232700	Glutamate, Kainate	314298	rat	2	10 µM	3				
232810	Glutamate, NMDA, Agonism	314451	rat	2	10 µM	-8				
232910	Glutamate, NMDA, Glycine	314253	rat	2	10 µM	6				
233000	Glutamate, NMDA, Phencyclidine	314178	rat	2	10 µM	2				
239610	Histamine H_1	314180	hum	2	10 µM	-16				

Note: Items meeting criteria for significance (≥50% stimulation or inhibition) are highlighted.
* Batch: Represents compounds tested concurrently in the same assay(s).
R=See Remarks (if any) at end of this section.
ham=Hamster; hum=Human

Experimental Results-DU011

Cat #	Assay Name	Batch*	Spec.	Rep.	Conc.	% Inh.	IC_{50}*	K_i	n_H	R
239710	Histamine H_2	314258	hum	2	10 µM	2				
239820	Histamine H_3	314302	hum	2	10 µM	13				
241000	Imidazoline I_2, Central	314181	rat	2	10 µM	-23				
243520	Interleukin IL-1	314186	mouse	2	10 µM	4				
250460	Leukotriene, Cysteinyl $CysLT_1$	314262	hum	2	10 µM	19				
251600	Melatonin MT_1	314266	hum	2	10 µM	7				
252610	Muscarinic M_1	314320	hum	2	10 µM	10				
252710	Muscarinic M_2	314183	hum	2	10 µM	5				
252810	Muscarinic M_3	314185	hum	2	10 µM	-3				
257010	Neuropeptide Y Y_1	314271	hum	2	10 µM	2				
257110	Neuropeptide Y Y_2	314303	hum	2	10 µM	-6				
258590	Nicotinic Acetylcholine	314188	hum	2	10 µM	22				
258700	Nicotinic Acetylcholine α1, Bungarotoxin	314189	hum	2	10 µM	8				
260130	Opiate δ_1 (OP1, DOP)	314191	hum	2	10 µM	10				
260210	Opiate κ(OP2, KOP)	314304	hum	2	10 µM	10				
260410	Opiate µ(OP3, MOP)	314194	hum	2	10 µM	1				
264500	Phorbol Ester	314195	mouse	2	10 µM	1				
265010	Platelet Activating Factor (PAF)	314197	hum	2	10 µM	-1				
265600	Potassium Channel [K_{ATP}]	314201	ham	2	10 µM	12				
265900	Potassium Channel hERG	314202	hum	2	10 µM	9				
268420	Prostanoid EP_4	314208	hum	2	10 µM	-4				
268700	Purinergic P_{2X}	314300	rabbit	2	10 µM	6				
268810	Purinergic P_{2Y}	314301	rat	2	10 µM	1				
270000	Rolipram	314203	rat	2	10 µM	3				
271110	Serotonin (5-Hydroxytryptamine) $5\text{-}HT_{1A}$	314275	hum	2	10 µM	11				
271700	Serotonin (5-Hydroxytryptamine) $5\text{-}HT_{2B}$	314205	hum	2	10 µM	19				
271910	Serotonin (5-Hydroxytryptamine) $5\text{-}HT_3$	314281	hum	2	10 µM	-7				
278110	Sigma σ_1	314095	hum	2	10 µM	9				
255520	Tachykinin NK_1	314269	hum	2	10 µM	7				
285900	Thyroid Hormone	314309	rat	2	10 µM	20				
220320	Transporter, Dopamine (DAT)	314094	hum	2	10 µM	-13				
226400	Transporter, GABA	314299	rat	2	10 µM	-4				
204410	Transporter, Norepinephrine (NET)	314086	hum	2	10 µM	58				
274030	Transporter, Serotonin (5-Hydroxytryptamine) (SERT)	314284	hum	2	10 µM	5				

Note: Items meeting criteria for significance (≥50% stimulation or inhibition) are highlighted.
* Batch: Represents compounds tested concurrently in the same assay(s).
R=See Remarks (if any) at end of this section.
ham=Hamster; hum=Human

Ricera LeadProfilingScreen Methods

■ 200510 Adenosine A_1

**Source:**	Human recombinant CHO cells	**Ligand:**	1.0 nM [^{3}H] DPCPX
**Vehicle:**	1% DMSO	**Non-Specific Ligand:**	100 µM R(-)-PIA
**Incubation Time/Temp:**	90 minutes @ 25°C	**Specific Binding:**	85% *
**Incubation Buffer:**	20 mM HEPES, pH 7.4, 10 mM $MgCl_2$, 100 mM NaCl	**Quantitation Method:**	Radioligand Binding
**Kd:**	1.40 nM *	**Significance Criteria:**	≥50% of max stimulation or inhibition
		**Bmax:**	2.70 pmole/mg Protein *

■ 200610 Adenosine A_{2A}

**Source:**	Human recombinant HEK-293 cells	**Ligand:**	0.050 µM [^{3}H] CGS-21680
**Vehicle:**	1% DMSO	**Non-Specific Ligand:**	50.0 µM NECA
**Incubation Time/Temp:**	90 minutes @ 25°C	**Specific Binding:**	85% *
**Incubation Buffer:**	50 mM Tris-HCl, pH 7.4, 10 mM $MgCl_2$, 1 mM EDTA, 2 U/mL Adenosine Deaminase	**Quantitation Method:**	Radioligand Binding
**Kd:**	0.064 µM *	**Significance Criteria:**	≥50% of max stimulation or inhibition
		**Bmax:**	7.0 pmole/mg Protein *

■ 200720 Adenosine A_3

**Source:**	Human recombinant CHO-K1 cells	**Ligand:**	0.50 nM [^{125}I] AB-MECA
**Vehicle:**	1% DMSO	**Non-Specific Ligand:**	1.0 µM IB-MECA
**Incubation Time/Temp:**	60 minutes @ 25°C	**Specific Binding:**	83% *
**Incubation Buffer:**	25 mM HEPES, pH 7.4, 5 mM $MgCl_2$, 1 mM $CaCl_2$, 0.1% BSA	**Quantitation Method:**	Radioligand Binding
**Kd:**	5.90 nM *	**Significance Criteria:**	≥50% of max stimulation or inhibition
		**Bmax:**	1.80 pmole/mg Protein *

■ 203100 Adrenergic α_{1A}

**Source:**	Wistar Rat submaxillary gland	**Ligand:**	0.25 nM [^{3}H] Prazosin
**Vehicle:**	1% DMSO	**Non-Specific Ligand:**	10.0 µM Phentolamine
**Incubation Time/Temp:**	60 minutes @ 25°C	**Specific Binding:**	90% *
**Incubation Buffer:**	50 mM Tris-HCl, pH 7.4, 0.5 mM EDTA	**Quantitation Method:**	Radioligand Binding
**Kd:**	0.17 nM *	**Significance Criteria:**	≥50% of max stimulation or inhibition
		**Bmax:**	0.18 pmole/mg Protein *

* Historical Values

Methods

■ 203200 Adrenergic α_{1B}

**Source:**	Wistar Rat liver	**Ligand:**	0.25 nM [^{3}H] Prazosin
**Vehicle:**	1% DMSO	**Non-Specific Ligand:**	10.0 µM Phentolamine
**Incubation Time/Temp:**	60 minutes @ 25°C	**Specific Binding:**	90% *
**Incubation Buffer:**	50 mM Tris-HCl, pH 7.4, 0. 5 mM EDTA	**Quantitation Method:**	Radioligand Binding
**Kd:**	0.31 nM *	**Significance Criteria:**	≥50% of max stimulation or inhibition
		**Bmax:**	0.18 pmole/mg Protein *

■ 203400 Adrenergic α_{1D}

**Source:**	Human recombinant HEK-293 cells	**Ligand:**	0.60 nM [^{3}H] Prazosin
**Vehicle:**	1% DMSO	**Non-Specific Ligand:**	10.0 µM Phentolamine
**Incubation Time/Temp:**	60 minutes @ 25°C	**Specific Binding:**	80% *
**Incubation Buffer:**	50 mM Tris-HCl, pH 7.4	**Quantitation Method:**	Radioligand Binding
**Kd:**	0.58 nM *	**Significance Criteria:**	≥50% of max stimulation or inhibition
		**Bmax:**	0.17 pmole/mg Protein *

■ 203620 Adrenergic α_{2A}

**Source:**	Human recombinant insect Sf9 cells	**Ligand:**	1.0 nM [^{3}H] MK-912
**Vehicle:**	1% DMSO	**Non-Specific Ligand:**	10.0 µM WB-4101
**Incubation Time/Temp:**	60 minutes @ 25°C	**Specific Binding:**	95% *
**Incubation Buffer:**	50 mM Tris-HCl, pH 7.4, 12.5 mM $MgCl_2$, 2 mM EDTA	**Quantitation Method:**	Radioligand Binding
**Kd:**	0.60 nM *	**Significance Criteria:**	≥50% of max stimulation or inhibition
		**Bmax:**	4.60 pmole/mg Protein *

■ 204010 Adrenergic β_1

**Source:**	Human recombinant CHO-K1 cells	**Ligand:**	0.030 nM [^{125}I] Cyanopindolol
**Vehicle:**	1% DMSO	**Non-Specific Ligand:**	100 µM S(-)-Propranolol
**Incubation Time/Temp:**	2 hours @ 25°C	**Specific Binding:**	95% *
**Incubation Buffer:**	50 mM Tris-HCl, pH 7.4, 1.4 mM Ascorbic Acid, 0.001% BSA, 5 mM EDTA, 1.5 mM $CaCl_2$, 120 mM NaCl	**Quantitation Method:**	Radioligand Binding
**Kd:**	0.041 nM *	**Significance Criteria:**	≥50% of max stimulation or inhibition
		**Bmax:**	0.072 pmole/mg Protein *

* Historical Values

Methods

■ 204110 Adrenergic β_2

**Source:**	Human recombinant CHO cells	**Ligand:**	0.20 nM [^{3}H] CGP-12177
**Vehicle:**	1% DMSO	**Non-Specific Ligand:**	10.0 µM ICI-118551
**Incubation Time/Temp:**	60 minutes @ 25°C	**Specific Binding:**	95% *
**Incubation Buffer:**	50 mM Tris-HCl, pH 7.4, 0.5 mM EDTA, 5.0 mM $MgCl_2$, 120 mM NaCl	**Quantitation Method:**	Radioligand Binding
**Kd:**	0.44 nM *	**Significance Criteria:**	≥50% of max stimulation or inhibition
		**Bmax:**	0.44 pmole/mg Protein *

■ 285010 Androgen (Testosterone) AR

**Source:**	Rat recombinant E. coli	**Ligand:**	1.50 nM [^{3}H] Mibolerone
**Vehicle:**	1% DMSO	**Non-Specific Ligand:**	10.0 µM Mibolerone
**Incubation Time/Temp:**	4 hours @ 4°C	**Specific Binding:**	90% *
**Incubation Buffer:**	50 mM Tris-HCl, pH 7.4, 0.8 M NaCl, 10% Glycerol, 2 mM Dithiothreitol, 0.1% BSA, 2% EtOH	**Quantitation Method:**	Radioligand Binding
**Kd:**	3.0 nM *	**Significance Criteria:**	≥50% of max stimulation or inhibition
		**Bmax:**	930 pmole/mg Protein *

■ 212510 Bradykinin B_1

**Source:**	Human IMR-90 cells	**Ligand:**	0.50 nM [^{3}H] (Des-Arg^{10})-Kallidin
**Vehicle:**	1% DMSO	**Non-Specific Ligand:**	10.0 µM (Des-Arg^9, Leu^8)-Bradykinin
**Incubation Time/Temp:**	60 minutes @ 25°C	**Specific Binding:**	80% *
**Incubation Buffer:**	20 mM HEPES, pH 7.4, 125 mM N-Methyl-D-glucamine, 5 mM KCl, 1 mM 1,10-Phenanthroline, 140 µg/ml Bacitracin	**Quantitation Method:**	Radioligand Binding
**Kd:**	0.17 nM *	**Significance Criteria:**	≥50% of max stimulation or inhibition
		**Bmax:**	0.55 pmole/mg Protein *

■ 212620 Bradykinin B_2

**Source:**	Human recombinant Chem-1 cells	**Ligand:**	0.50 nM [^{3}H] Bradykinin
**Vehicle:**	1% DMSO	**Non-Specific Ligand:**	5.0 µM Bradykinin
**Incubation Time/Temp:**	60 minutes @ 25°C	**Specific Binding:**	90% *
**Incubation Buffer:**	50 mM HEPES, pH 7.4, 0.2% BSA, 1 mM $CaCl_2$, 5 mM $MgCl_2$	**Quantitation Method:**	Radioligand Binding
**Kd:**	0.85 nM *	**Significance Criteria:**	≥50% of max stimulation or inhibition
		**Bmax:**	9.40 pmole/mg Protein *

* Historical Values

Methods

■ 214510 Calcium Channel L-Type, Benzothiazepine

**Source:**	Wistar Rat brain	**Ligand:**	2.0 nM [^{3}H] Diltiazem
**Vehicle:**	1% DMSO	**Non-Specific Ligand:**	10.0 µM Diltiazem
**Incubation Time/Temp:**	3 hours @ 4°C	**Specific Binding:**	73% *
**Incubation Buffer:**	50 mM Tris-HCl, pH 7.4, 0.1% BSA	**Quantitation Method:**	Radioligand Binding
**Kd:**	0.016 µM *	**Significance Criteria:**	≥50% of max stimulation or inhibition
		**Bmax:**	0.21 pmole/mg Protein *

■ 214600 Calcium Channel L-Type, Dihydropyridine

**Source:**	Wistar Rat cerebral cortex	**Ligand:**	0.10 nM [^{3}H] Nitrendipine
**Vehicle:**	1% DMSO	**Non-Specific Ligand:**	1.0 µM Nifedipine
**Incubation Time/Temp:**	90 minutes @ 25°C	**Specific Binding:**	91% *
**Incubation Buffer:**	50 mM Tris-HCl, pH 7.4	**Quantitation Method:**	Radioligand Binding
**Kd:**	0.18 nM *	**Significance Criteria:**	≥50% of max stimulation or inhibition
		**Bmax:**	0.23 pmole/mg Protein *

■ 216000 Calcium Channel N-Type

**Source:**	Wistar Rat frontal brain	**Ligand:**	10 pM [^{125}I] ω-Conotoxin GVIA
**Vehicle:**	1% DMSO	**Non-Specific Ligand:**	0.10 µM ω-Conotoxin GVIA
**Incubation Time/Temp:**	30 minutes @ 4°C	**Specific Binding:**	96% *
**Incubation Buffer:**	20 mM Tris-HCl, pH 7.4, 0.5% BSA	**Quantitation Method:**	Radioligand Binding
**Kd:**	0.051 nM *	**Significance Criteria:**	≥50% of max stimulation or inhibition
		**Bmax:**	0.88 pmole/mg Protein *

■ 217030 Cannabinoid CB_1

**Source:**	Human recombinant Chem-1 cells	**Ligand:**	2.0 nM [^{3}H] SR141716A
**Vehicle:**	1% DMSO	**Non-Specific Ligand:**	10.0 µM R(+)-WIN-55,212-2
**Incubation Time/Temp:**	90 minutes @ 37°C	**Specific Binding:**	70% *
**Incubation Buffer:**	50 mM HEPES, pH 7.4, 5 mM $MgCl_2$, 1 mM $CaCl_2$, 0.2% BSA	**Quantitation Method:**	Radioligand Binding
**Kd:**	5.90 nM *	**Significance Criteria:**	≥50% of max stimulation or inhibition
		**Bmax:**	15.0 pmole/mg Protein *

* Historical Values

Methods

■ 219500 Dopamine D_1

**Source:**	Human recombinant CHO cells	**Ligand:**	1.40 nM [^{3}H] SCH-23390
**Vehicle:**	1% DMSO	**Non-Specific Ligand:**	10.0 µM (+)-Butaclamol
**Incubation Time/Temp:**	2 hours @ 37°C	**Specific Binding:**	90% *
**Incubation Buffer:**	50 mM Tris-HCl, pH 7.4, 1.4 mM Ascorbic Acid, 0.001% BSA, 150 mM NaCl	**Quantitation Method:**	Radioligand Binding
**Kd:**	1.40 nM *	**Significance Criteria:**	≥50% of max stimulation or inhibition
		**Bmax:**	0.63 pmole/mg Protein *

■ 219700 Dopamine D_{2S}

**Source:**	Human recombinant CHO cells	**Ligand:**	0.16 nM [^{3}H] Spiperone
**Vehicle:**	1% DMSO	**Non-Specific Ligand:**	10.0 µM Haloperidol
**Incubation Time/Temp:**	2 hours @ 25°C	**Specific Binding:**	90% *
**Incubation Buffer:**	50 mM Tris-HCl, pH 7.4, 1.4 mM Ascorbic Acid, 0.001% BSA, 150 mM NaCl	**Quantitation Method:**	Radioligand Binding
**Kd:**	0.090 nM *	**Significance Criteria:**	≥50% of max stimulation or inhibition
		**Bmax:**	1.60 pmole/mg Protein *

■ 219800 Dopamine D_3

**Source:**	Human recombinant CHO cells	**Ligand:**	0.70 nM [^{3}H] Spiperone
**Vehicle:**	1% DMSO	**Non-Specific Ligand:**	25.0 µM S(-)-Sulpiride
**Incubation Time/Temp:**	2 hours @ 37°C	**Specific Binding:**	85% *
**Incubation Buffer:**	50 mM Tris-HCl, pH 7.4, 1.4 mM Ascorbic Acid, 0.001% BSA, 150 mM NaCl	**Quantitation Method:**	Radioligand Binding
**Kd:**	0.36 nM *	**Significance Criteria:**	≥50% of max stimulation or inhibition
		**Bmax:**	1.10 pmole/mg Protein *

■ 219900 Dopamine $D_{4.2}$

**Source:**	Human recombinant CHO-K1 cells	**Ligand:**	0.50 nM [^{3}H] Spiperone
**Vehicle:**	1% DMSO	**Non-Specific Ligand:**	10.0 µM Haloperidol
**Incubation Time/Temp:**	2 hours @ 25°C	**Specific Binding:**	90% *
**Incubation Buffer:**	50 mM Tris-HCl, pH 7.4, 1.4 mM Ascorbic Acid, 0.001% BSA, 150 mM NaCl	**Quantitation Method:**	Radioligand Binding
**Kd:**	0.32 nM *	**Significance Criteria:**	≥50% of max stimulation or inhibition
		**Bmax:**	0.55 pmole/mg Protein *

* Historical Values

Methods

■ 224010 Endothelin ET_A

**Source:**	Human recombinant CHO-K1 cells	**Ligand:**	0.030 nM [^{125}I] Endothelin-1
**Vehicle:**	1% DMSO	**Non-Specific Ligand:**	0.10 µM Endothelin-1
**Incubation Time/Temp:**	2 hours @ 37°C	**Specific Binding:**	90% *
**Incubation Buffer:**	50 mM Tris-HCl, pH 7.4, 0.1% BSA, 0.5 mM $CaCl_2$, 0.05% Tween-20	**Quantitation Method:**	Radioligand Binding
**Kd:**	0.048 nM *	**Significance Criteria:**	≥50% of max stimulation or inhibition
		**Bmax:**	0.35 pmole/mg Protein *

■ 224110 Endothelin ET_B

**Source:**	Human recombinant CHO-K1 cells	**Ligand:**	0.10 nM [^{125}I] Endothelin-1
**Vehicle:**	1% DMSO	**Non-Specific Ligand:**	0.10 µM Endothelin-1
**Incubation Time/Temp:**	2 hours @ 25°C	**Specific Binding:**	75% *
**Incubation Buffer:**	50 mM HEPES, pH 7.4, 1 mM $CaCl_2$, 5 mM $MgCl_2$, 0.5% BSA	**Quantitation Method:**	Radioligand Binding
**Kd:**	0.085 nM *	**Significance Criteria:**	≥50% of max stimulation or inhibition
		**Bmax:**	4.30 pmole/mg Protein *

■ 225510 Epidermal Growth Factor (EGF)

**Source:**	Human A431 cells	**Ligand:**	0.080 nM [^{125}I] EGF (human)
**Vehicle:**	1% DMSO	**Non-Specific Ligand:**	0.10 µM EGF (human)
**Incubation Time/Temp:**	60 minutes @ 25°C	**Specific Binding:**	90% *
**Incubation Buffer:**	50 mM HEPES, pH 7.7, 0.1% BSA, 1.2 mM $CaCl_2$, 5 mM KCl, 1.2 mM $MgSO_4$, 138 mM NaCl	**Quantitation Method:**	Radioligand Binding
**Kd:**	0.17 nM *	**Significance Criteria:**	≥50% of max stimulation or inhibition
		**Bmax:**	5.50 pmole/mg Protein *

■ 226010 Estrogen ERα

**Source:**	Human recombinant insect Sf9 cells	**Ligand:**	0.50 nM [^{3}H] Estradiol
**Vehicle:**	1% DMSO	**Non-Specific Ligand:**	1.0 µM Diethylstilbestrol
**Incubation Time/Temp:**	2 hours @ 25°C	**Specific Binding:**	85% *
**Incubation Buffer:**	10 mM Tris-HCl, pH 7.4, 0.1% BSA, 10% Glycerol, 1 mM DTT	**Quantitation Method:**	Radioligand Binding
**Kd:**	0.20 nM *	**Significance Criteria:**	≥50% of max stimulation or inhibition
		**Bmax:**	1400 pmole/mg Protein *

* Historical Values

Methods

226600 GABA$_A$, Flunitrazepam, Central

**Source:**	Wistar Rat brain (minus cerebellum)	**Ligand:**	1.0 nM [^{3}H] Flunitrazepam
**Vehicle:**	1% DMSO	**Non-Specific Ligand:**	10.0 µM Diazepam
**Incubation Time/Temp:**	60 minutes @ 25°C	**Specific Binding:**	91% *
**Incubation Buffer:**	50 mM Phosphate Buffer, pH 7.4	**Quantitation Method:**	Radioligand Binding
**Kd:**	4.40 nM *	**Significance Criteria:**	≥50% of max stimulation or inhibition
		**Bmax:**	1.20 pmole/mg Protein *

226500 GABA$_A$, Muscimol, Central

**Source:**	Wistar Rat brain (minus cerebellum)	**Ligand:**	1.0 nM [^{3}H] Muscimol
**Vehicle:**	1% DMSO	**Non-Specific Ligand:**	0.10 µM Muscimol
**Incubation Time/Temp:**	10 minutes @ 4°C	**Specific Binding:**	90% *
**Incubation Buffer:**	50 mM Tris-HCl, pH 7.4	**Quantitation Method:**	Radioligand Binding
**Kd:**	3.80 nM *	**Significance Criteria:**	≥50% of max stimulation or inhibition
		**Bmax:**	1.80 pmole/mg Protein *

228610 GABA$_{B1A}$

**Source:**	Human recombinant CHO cells	**Ligand:**	4.0 nM [^{3}H] CGP-54626
**Vehicle:**	1% DMSO	**Non-Specific Ligand:**	3.0 mM GABA
**Incubation Time/Temp:**	3 hours @ 25°C	**Specific Binding:**	90% *
**Incubation Buffer:**	50 mM Tris-HCl, pH 7.4, 2.5 mM $CaCl_2$, 0.1% BSA	**Quantitation Method:**	Radioligand Binding
**Kd:**	3.30 nM *	**Significance Criteria:**	≥50% of max stimulation or inhibition
		**Bmax:**	48.0 pmole/mg Protein *

232030 Glucocorticoid

**Source:**	Human recombinant Insect cells	**Ligand:**	5.0 nM [^{3}H] Dexamethasone
**Vehicle:**	1% DMSO	**Non-Specific Ligand:**	10.0 µM Dexamethasone
**Incubation Time/Temp:**	1 day @ 4°C	**Specific Binding:**	97% *
**Incubation Buffer:**	5 mM KH_2PO_4, 8 mM $Na_2HPO_4 \cdot 12H_2O$, pH7.4, 137 mM NaCl, 2.7 mM KCl, 0.2% BSA	**Quantitation Method:**	Radioligand Binding
**Kd:**	4.60 nM *	**Significance Criteria:**	≥50% of max stimulation or inhibition
		**Bmax:**	1.0 pmole/mg *

* Historical Values

Methods

■ 232700 Glutamate, Kainate

**Source:**	Wistar Rat brain (minus cerebellum)	**Ligand:**	5.0 nM [^{3}H] Kainic acid
**Vehicle:**	1% DMSO	**Non-Specific Ligand:**	1.0 mM L-Glutamic acid
**Incubation Time/Temp:**	60 minutes @ 4°C	**Specific Binding:**	80% *
**Incubation Buffer:**	50 mM Tris-HCl, pH 7.4	**Quantitation Method:**	Radioligand Binding
**Kd:**	0.012 µM *	**Significance Criteria:**	≥50% of max stimulation or inhibition
		**Bmax:**	0.35 pmole/mg Protein *

■ 232810 Glutamate, NMDA, Agonism

**Source:**	Wistar Rat cerebral cortex	**Ligand:**	2.0 nM [^{3}H] CGP-39653
**Vehicle:**	1% DMSO	**Non-Specific Ligand:**	1.0 mM L-Glutamic acid
**Incubation Time/Temp:**	20 minutes @ 4°C	**Specific Binding:**	70% *
**Incubation Buffer:**	50 mM Tris-HCl, pH 7.4	**Quantitation Method:**	Radioligand Binding
**Kd:**	0.019 µM *	**Significance Criteria:**	≥50% of max stimulation or inhibition
		**Bmax:**	2.30 pmole/mg Protein *

■ 232910 Glutamate, NMDA, Glycine

**Source:**	Wistar Rat cerebral cortex	**Ligand:**	0.33 nM [^{3}H] MDL 105,519
**Vehicle:**	1% DMSO	**Non-Specific Ligand:**	10.0 µM MDL 105,519
**Incubation Time/Temp:**	30 minutes @ 4°C	**Specific Binding:**	85% *
**Incubation Buffer:**	50 mM HEPES, pH 7.7	**Quantitation Method:**	Radioligand Binding
**Kd:**	6.0 nM *	**Significance Criteria:**	≥50% of max stimulation or inhibition
		**Bmax:**	3.70 pmole/mg Protein *

■ 233000 Glutamate, NMDA, Phencyclidine

**Source:**	Wistar Rat cerebral cortex	**Ligand:**	4.0 nM [^{3}H] TCP
**Vehicle:**	1% DMSO	**Non-Specific Ligand:**	1.0 µM Dizocilpine ((+)-MK-801)
**Incubation Time/Temp:**	45 minutes @ 25°C	**Specific Binding:**	94% *
**Incubation Buffer:**	10 mM Tris-HCl, pH 7.4	**Quantitation Method:**	Radioligand Binding
**Kd:**	8.40 nM *	**Significance Criteria:**	≥50% of max stimulation or inhibition
		**Bmax:**	0.78 pmole/mg Protein *

* Historical Values

Methods

■ 239610 Histamine H_1

**Source:**	Human recombinant CHO-K1 cells	**Ligand:**	1.20 nM [^{3}H] Pyrilamine
**Vehicle:**	1% DMSO	**Non-Specific Ligand:**	1.0 µM Pyrilamine
**Incubation Time/Temp:**	3 hours @ 25°C	**Specific Binding:**	94% *
**Incubation Buffer:**	50 mM Tris-HCl, pH 7.4, 2 mM $MgCl_2$, 100 mM NaCl, 250 mM Sucrose	**Quantitation Method:**	Radioligand Binding
**Kd:**	1.10 nM *	**Significance Criteria:**	≥50% of max stimulation or inhibition
		**Bmax:**	6.70 pmole/mg Protein *

■ 239710 Histamine H_2

**Source:**	Human recombinant CHO-K1 cells	**Ligand:**	0.10 nM [^{125}I] Aminopotentidine
**Vehicle:**	1% DMSO	**Non-Specific Ligand:**	3.0 µM Tiotidine
**Incubation Time/Temp:**	2 hours @ 25°C	**Specific Binding:**	90% *
**Incubation Buffer:**	50 mM Phosphate, pH 7.4	**Quantitation Method:**	Radioligand Binding
**Kd:**	0.45 nM *	**Significance Criteria:**	≥50% of max stimulation or inhibition
		**Bmax:**	6.90 pmole/mg Protein *

■ 239820 Histamine H_3

**Source:**	Human recombinant CHO-K1 cells	**Ligand:**	0.40 nM [^{3}H] N-α-Methylhistamine (NAMH)
**Vehicle:**	1% DMSO	**Non-Specific Ligand:**	1.0 µM R(-)-α-Methylhistamine (RAMH)
**Incubation Time/Temp:**	2 hours @ 25°C	**Specific Binding:**	90% *
**Incubation Buffer:**	50 mM Tris-HCl, pH 7.4, 5 mM $MgCl_2$, 0.1% BSA	**Quantitation Method:**	Radioligand Binding
**Kd:**	0.38 nM *	**Significance Criteria:**	≥50% of max stimulation or inhibition
		**Bmax:**	2.0 pmole/mg Protein *

■ 241000 Imidazoline I_2, Central

**Source:**	Wistar Rat cerebral cortex	**Ligand:**	2.0 nM [^{3}H] Idazoxan
**Vehicle:**	1% DMSO	**Non-Specific Ligand:**	1.0 µM Idazoxan
**Incubation Time/Temp:**	30 minutes @ 25°C	**Specific Binding:**	85% *
**Incubation Buffer:**	50 mM Tris-HCl, pH 7.4, 0.5 mM EDTA	**Quantitation Method:**	Radioligand Binding
**Kd:**	4.0 nM *	**Significance Criteria:**	≥50% of max stimulation or inhibition
		**Bmax:**	0.14 pmole/mg Protein *

* Historical Values

Methods

■ 243520 Interleukin IL-1

**Source:**	Mouse 3T3-SWISS cells	**Ligand:**	0.10 nM [^{125}I] Interleukin-1β
**Vehicle:**	1% DMSO	**Non-Specific Ligand:**	10.0 µM Interleukin-1β
**Incubation Time/Temp:**	2 hours @ 37°C	**Specific Binding:**	80% *
**Incubation Buffer:**	RPMI 1640, 20 mM HEPES, pH 7.4, 0.1% Sodium Azide, 1% BSA	**Quantitation Method:**	Radioligand Binding
**Kd:**	0.25 nM *	**Significance Criteria:**	≥50% of max stimulation or inhibition
		**Bmax:**	820 R/cell Protein *

■ 250460 Leukotriene, Cysteinyl $CysLT_1$

**Source:**	Human recombinant CHO-K1 cells	**Ligand:**	0.30 nM [^{3}H] LTD_4
**Vehicle:**	1% DMSO	**Non-Specific Ligand:**	0.30 µM LTD_4
**Incubation Time/Temp:**	30 minutes @ 25°C	**Specific Binding:**	93% *
**Incubation Buffer:**	50 mM Tris-HCl, pH 7.4, 5 mM $CaCl_2$, 5 mM $MgCl_2$, 100 µg/ml Bacitracin, 1 mM Benzamidine, 0.1 mM PMSF	**Quantitation Method:**	Radioligand Binding
**Kd:**	0.21 nM *	**Significance Criteria:**	≥50% of max stimulation or inhibition
		**Bmax:**	3.0 pmole/mg Protein *

■ 251600 Melatonin MT_1

**Source:**	Human recombinant CHO-K1 cells	**Ligand:**	0.050 nM [^{125}I] 2-Iodomelatonin
**Vehicle:**	1% DMSO	**Non-Specific Ligand:**	1.0 µM 6-Chloromelatonin
**Incubation Time/Temp:**	3 hours @ 25°C	**Specific Binding:**	97% *
**Incubation Buffer:**	25 mM HEPES, pH 7.4, 5 mM $MgCl_2$, 1 mM $CaCl_2$, 0.5% BSA	**Quantitation Method:**	Radioligand Binding
**Kd:**	0.054 nM *	**Significance Criteria:**	≥50% of max stimulation or inhibition
		**Bmax:**	3.50 pmole/mg Protein *

■ 252610 Muscarinic M_1

**Source:**	Human recombinant CHO-K1 cells	**Ligand:**	0.80 nM [^{3}H] N-Methylscopolamine
**Vehicle:**	1% DMSO	**Non-Specific Ligand:**	1.0 µM Atropine
**Incubation Time/Temp:**	2 hours @ 25°C	**Specific Binding:**	95% *
**Incubation Buffer:**	50 mM Tris-HCl, pH 7.4, 10 mM $MgCl_2$, 1 mM EDTA	**Quantitation Method:**	Radioligand Binding
**Kd:**	0.26 nM *	**Significance Criteria:**	≥50% of max stimulation or inhibition
		**Bmax:**	2.0 pmole/mg Protein *

* Historical Values

Methods

■ 252710 Muscarinic M_2

**Source:**	Human recombinant CHO-K1 cells	**Ligand:**	0.80 nM [^{3}H] N-Methylscopolamine
**Vehicle:**	1% DMSO	**Non-Specific Ligand:**	1.0 µM Atropine
**Incubation Time/Temp:**	2 hours @ 25°C	**Specific Binding:**	95% *
**Incubation Buffer:**	50 mM Tris-HCl, pH 7.4, 10 mM $MgCl_2$, 1 mM EDTA	**Quantitation Method:**	Radioligand Binding
**Kd:**	0.58 nM *	**Significance Criteria:**	≥50% of max stimulation or inhibition
		**Bmax:**	5.10 pmole/mg Protein *

■ 252810 Muscarinic M_3

**Source:**	Human recombinant CHO-K1 cells	**Ligand:**	0.80 nM [^{3}H] N-Methylscopolamine
**Vehicle:**	1% DMSO	**Non-Specific Ligand:**	1.0 µM Atropine
**Incubation Time/Temp:**	2 hours @ 25°C	**Specific Binding:**	95% *
**Incubation Buffer:**	50 mM Tris-HCl, pH 7.4, 10 mM $MgCl_2$, 1 mM EDTA	**Quantitation Method:**	Radioligand Binding
**Kd:**	0.75 nM *	**Significance Criteria:**	≥50% of max stimulation or inhibition
		**Bmax:**	5.40 pmole/mg Protein *

■ 257010 Neuropeptide Y Y_1

**Source:**	Human SK-N-MC cells	**Ligand:**	0.015 nM [^{125}I] Peptide YY
**Vehicle:**	1% DMSO	**Non-Specific Ligand:**	1.0 µM Neuropeptide Y (human, rat)
**Incubation Time/Temp:**	60 minutes @ 37°C	**Specific Binding:**	80% *
**Incubation Buffer:**	25 mM HEPES, pH 7.4, 1 mM $MgCl_2$, 2.5 mM $CaCl_2$, 0.1% BSA, 0.01% Bacitracin	**Quantitation Method:**	Radioligand Binding
**Kd:**	0.24 nM *	**Significance Criteria:**	≥50% of max stimulation or inhibition
		**Bmax:**	0.58 pmole/mg protein *

■ 257110 Neuropeptide Y Y_2

**Source:**	Human KAN-TS cells	**Ligand:**	10 pM [^{125}I] Peptide YY
**Vehicle:**	1% DMSO	**Non-Specific Ligand:**	1.0 µM Neuropeptide Y (13-36) (porcine)
**Incubation Time/Temp:**	2 hours @ 37°C	**Specific Binding:**	90% *
**Incubation Buffer:**	25 mM HEPES, pH 7.4, 2.5 mM $CaCl_2$, 1 mM $MgCl_2$, 0.1% Bacitracin	**Quantitation Method:**	Radioligand Binding
**Kd:**	0.012 nM *	**Significance Criteria:**	≥50% of max stimulation or inhibition
		**Bmax:**	0.5 pmole/mg Protein *

* Historical Values

Methods

■ 258590 Nicotinic Acetylcholine

**Source:**	Human IMR-32 cells	**Ligand:**	0.10 nM [^{125}I] Epibatidine
**Vehicle:**	1% DMSO	**Non-Specific Ligand:**	300 μM (-)-Nicotine
**Incubation Time/Temp:**	60 minutes @ 25°C	**Specific Binding:**	97% *
**Incubation Buffer:**	20 mM HEPES, pH 7.5, 150 mM NaCl, 1.5 mM KCl, 2 mM $CaCl_2$, 1 mM $MgSO_4$.	**Quantitation Method:**	Radioligand Binding
**Kd:**	0.22 nM *	**Significance Criteria:**	≥50% of max stimulation or inhibition
		**Bmax:**	0.46 pmole/mg Protein *

■ 258700 Nicotinic Acetylcholine α1, Bungarotoxin

**Source:**	Human RD cells	**Ligand:**	0.60 nM [^{125}I] α-Bungarotoxin
**Vehicle:**	1% DMSO	**Non-Specific Ligand:**	1.0 μM α-Bungarotoxin
**Incubation Time/Temp:**	2 hours @ 25°C	**Specific Binding:**	85% *
**Incubation Buffer:**	150 mM NaCl, 4 mM KCl, 2.3 mM $CaCl_2$	**Quantitation Method:**	Radioligand Binding
**Kd:**	1.10 nM *	**Significance Criteria:**	≥50% of max stimulation or inhibition
		**Bmax:**	1.0 pmole/mg Protein *

■ 260130 Opiate δ_1 (OP1, DOP)

**Source:**	Human recombinant HEK-293 cells	**Ligand:**	1.30 nM [^{3}H] Naltrindole
**Vehicle:**	1% DMSO	**Non-Specific Ligand:**	1.0 μM Naltrindole
**Incubation Time/Temp:**	60 minutes @ 25°C	**Specific Binding:**	95% *
**Incubation Buffer:**	50 mM Tris-HCl, pH 7.4, 1 mM EDTA, 10 mM $MgCl_2$	**Quantitation Method:**	Radioligand Binding
**Kd:**	0.27 nM *	**Significance Criteria:**	≥50% of max stimulation or inhibition
		**Bmax:**	7.60 pmole/mg Protein *

■ 260210 Opiate κ(OP2, KOP)

**Source:**	Human recombinant HEK-293 cells	**Ligand:**	0.60 nM [^{3}H] Diprenorphine
**Vehicle:**	1% DMSO	**Non-Specific Ligand:**	10.0 μM Naloxone
**Incubation Time/Temp:**	60 minutes @ 25°C	**Specific Binding:**	90% *
**Incubation Buffer:**	50 mM Tris-HCl, pH 7.4	**Quantitation Method:**	Radioligand Binding
**Kd:**	0.40 nM *	**Significance Criteria:**	≥50% of max stimulation or inhibition
		**Bmax:**	1.10 pmole/mg Protein *

* Historical Values

Methods

■ 260410 Opiate μ(OP3, MOP)

**Source:**	Human recombinant CHO-K1 cells	**Ligand:**	0.60 nM [^{3}H] Diprenorphine
**Vehicle:**	1% DMSO	**Non-Specific Ligand:**	10.0 μM Naloxone
**Incubation Time/Temp:**	60 minutes @ 25°C	**Specific Binding:**	90% *
**Incubation Buffer:**	50 mM Tris-HCl, pH 7.4	**Quantitation Method:**	Radioligand Binding
**Kd:**	0.41 nM *	**Significance Criteria:**	≥50% of max stimulation or inhibition
		**Bmax:**	3.80 pmole/mg Protein *

■ 264500 Phorbol Ester

**Source:**	ICR Mouse brain	**Ligand:**	3.0 nM [^{3}H] PDBu
**Vehicle:**	1% DMSO	**Non-Specific Ligand:**	1.0 μM PDBu
**Incubation Time/Temp:**	60 minutes @ 25°C	**Specific Binding:**	80% *
**Incubation Buffer:**	20 mM Tris-HCl, pH 7.4, 5 mM $CaCl_2$	**Quantitation Method:**	Radioligand Binding
**Kd:**	8.70 nM *	**Significance Criteria:**	≥50% of max stimulation or inhibition
		**Bmax:**	26.0 pmole/mg Protein *

■ 265010 Platelet Activating Factor (PAF)

**Source:**	Human platelets	**Ligand:**	0.12 nM [^{3}H] PAF
**Vehicle:**	1% DMSO	**Non-Specific Ligand:**	1.0 μM PAF
**Incubation Time/Temp:**	3 hours @ 25°C	**Specific Binding:**	90% *
**Incubation Buffer:**	50 mM Tris-HCl, pH 7.4, 100 mM KCl, 5 mM EDTA, 5 mM $MgCl_2$, 0.25% BSA	**Quantitation Method:**	Radioligand Binding
**Kd:**	0.13 nM *	**Significance Criteria:**	≥50% of max stimulation or inhibition
		**Bmax:**	120 R/cell *

■ 265600 Potassium Channel [K_{ATP}]

**Source:**	Hamster pancreatic HIT-T15 beta cells	**Ligand:**	5.0 nM [^{3}H] Glyburide
**Vehicle:**	1% DMSO	**Non-Specific Ligand:**	1.0 μM Glyburide
**Incubation Time/Temp:**	2 hours @ 25°C	**Specific Binding:**	90% *
**Incubation Buffer:**	50 mM MOPS, pH 7.4, 0.1 mM $CaCl_2$	**Quantitation Method:**	Radioligand Binding
**Kd:**	0.64 nM *	**Significance Criteria:**	≥50% of max stimulation or inhibition
		**Bmax:**	1.0 pmole/mg Protein *

* Historical Values

Methods

■ 265900 Potassium Channel hERG

**Source:**	Human recombinant HEK-293 cells	**Ligand:**	1.50 nM [^{3}H] Astemizole
**Vehicle:**	1% DMSO	**Non-Specific Ligand:**	10.0 µM Astemizole
**Incubation Time/Temp:**	60 minutes @ 25°C	**Specific Binding:**	90% *
**Incubation Buffer:**	10 mM HEPES, pH 7.4, 0.1% BSA, 5 mM KCl, 0.8 mM $MgCl_2$, 130 mM NaCl, 1 mM EGTA, 10 mM Glucose	**Quantitation Method:**	Radioligand Binding
**Kd:**	6.80 nM *	**Significance Criteria:**	≥50% of max stimulation or inhibition
		**Bmax:**	6.30 pmole/mg Protein *

■ 268420 Prostanoid EP_4

**Source:**	Human recombinant Chem-1 cells	**Ligand:**	1.0 nM [^{3}H] Prostaglandin E_2 (PGE_2)
**Vehicle:**	1% DMSO	**Non-Specific Ligand:**	10.0 µM Prostaglandin E_2 (PGE_2)
**Incubation Time/Temp:**	2 hours @ 25°C	**Specific Binding:**	90% *
**Incubation Buffer:**	10 mM MES, pH 6.0, 1 mM EDTA, 10 mM $MgCl_2$	**Quantitation Method:**	Radioligand Binding
**Kd:**	0.69 nM *	**Significance Criteria:**	≥50% of max stimulation or inhibition
		**Bmax:**	4.30 pmole/mg Protein *

■ 268700 Purinergic P_{2X}

**Source:**	New Zealand derived albino Rabbit urinary bladder	**Ligand:**	8.0 nM [^{3}H] α, β-Methylene-ATP
**Vehicle:**	1% DMSO	**Non-Specific Ligand:**	100 µM β, γ-Methylene ATP
**Incubation Time/Temp:**	30 minutes @ 25°C	**Specific Binding:**	80% *
**Incubation Buffer:**	50 mM Tris-HCl, pH 7.4	**Quantitation Method:**	Radioligand Binding
**Kd1:**	2.20 nM *	**Significance Criteria:**	≥50% of max stimulation or inhibition
**Kd2:**	2.20 µM *	**Bmax1:**	2.0 pmole/mg Protein *
		**Bmax2:**	790 pmole/mg Protein *

■ 268810 Purinergic P_{2Y}

**Source:**	Wistar Rat brain	**Ligand:**	0.10 nM [^{35}S] ATP-αS
**Vehicle:**	1% DMSO	**Non-Specific Ligand:**	10.0 µM ADP-βS
**Incubation Time/Temp:**	60 minutes @ 25°C	**Specific Binding:**	87% *
**Incubation Buffer:**	50 mM Tris-HCl, pH 7.4	**Quantitation Method:**	Radioligand Binding
**Kd:**	0.015 µM *	**Significance Criteria:**	≥50% of max stimulation or inhibition
		**Bmax:**	16.0 pmole/mg Protein *

* Historical Values

Methods

■ 270000 Rolipram

**Source:**	Wistar Rat brain	**Ligand:**	1.80 nM [^{3}H] Rolipram
**Vehicle:**	1% DMSO	**Non-Specific Ligand:**	10.0 µM Rolipram
**Incubation Time/Temp:**	60 minutes @ 4°C	**Specific Binding:**	90% *
**Incubation Buffer:**	50 mM Tris-HCl, pH 7.4	**Quantitation Method:**	Radioligand Binding
**Kd:**	1.0 nM *	**Significance Criteria:**	≥50% of max stimulation or inhibition
		**Bmax:**	0.31 pmole/mg Protein *

■ 271110 Serotonin (5-Hydroxytryptamine) 5-HT_{1A}

**Source:**	Human recombinant CHO-K1 cells	**Ligand:**	1.50 nM [^{3}H] 8-OH-DPAT
**Vehicle:**	1% DMSO	**Non-Specific Ligand:**	10.0 µM Metergoline
**Incubation Time/Temp:**	60 minutes @ 25°C	**Specific Binding:**	75% *
**Incubation Buffer:**	50 mM Tris-HCl, pH 7.4, 0.1% Ascorbic Acid, 0.5 mM EDTA, 10 mM $MgSO_4$	**Quantitation Method:**	Radioligand Binding
**Kd:**	2.0 nM *	**Significance Criteria:**	≥50% of max stimulation or inhibition
		**Bmax:**	1.30 pmole/mg Protein *

■ 271700 Serotonin (5-Hydroxytryptamine) 5-HT_{2B}

**Source:**	Human recombinant CHO-K1 cells	**Ligand:**	1.20 nM [^{3}H] Lysergic acid diethylamide (LSD)
**Vehicle:**	1% DMSO	**Non-Specific Ligand:**	10.0 µM Serotonin (5-HT)
**Incubation Time/Temp:**	60 minutes @ 37°C	**Specific Binding:**	80% *
**Incubation Buffer:**	50 mM Tris-HCl, pH 7.4, 4 mM $CaCl_2$, 0.1% Ascorbic Acid	**Quantitation Method:**	Radioligand Binding
**Kd:**	2.10 nM *	**Significance Criteria:**	≥50% of max stimulation or inhibition
		**Bmax:**	1.10 pmole/mg Protein *

■ 271910 Serotonin (5-Hydroxytryptamine) 5-HT_3

**Source:**	Human recombinant HEK-293 cells	**Ligand:**	0.69 nM [^{3}H] GR-65630
**Vehicle:**	1% DMSO	**Non-Specific Ligand:**	10.0 µM MDL 72222
**Incubation Time/Temp:**	60 minutes @ 25°C	**Specific Binding:**	90% *
**Incubation Buffer:**	50 mM Tris-HCl, pH 7.4, 1 mM EDTA, 5 mM $MgCl_2$	**Quantitation Method:**	Radioligand Binding
**Kd:**	0.20 nM *	**Significance Criteria:**	≥50% of max stimulation or inhibition
		**Bmax:**	11.0 pmole/mg Protein *

* Historical Values

Methods

■ 278110 Sigma σ_1

**Source:**	Human Jurkat cells	**Ligand:**	8.0 nM [^{3}H] Haloperidol
**Vehicle:**	1% DMSO	**Non-Specific Ligand:**	10.0 µM Haloperidol
**Incubation Time/Temp:**	4 hours @ 25°C	**Specific Binding:**	80% *
**Incubation Buffer:**	5 mM Potassium Phosphate, pH 7.5	**Quantitation Method:**	Radioligand Binding
**Kd:**	5.80 nM *	**Significance Criteria:**	≥50% of max stimulation or inhibition
		**Bmax:**	0.71 pmole/mg Protein *

■ 255520 Tachykinin NK_1

**Source:**	Human recombinant CHO cells	**Ligand:**	0.80 nM [^{3}H] Substance P
**Vehicle:**	1% DMSO	**Non-Specific Ligand:**	10.0 µM L-703,606
**Incubation Time/Temp:**	90 minutes @ 4°C	**Specific Binding:**	90% *
**Incubation Buffer:**	20 mM HEPES, pH 7.4, 1 mM $MnCl_2$, 0.1% BSA	**Quantitation Method:**	Radioligand Binding
**Kd:**	2.10 nM *	**Significance Criteria:**	≥50% of max stimulation or inhibition
		**Bmax:**	1.70 pmole/mg Protein *

■ 285900 Thyroid Hormone

**Source:**	Wistar Rat liver	**Ligand:**	0.030 nM [^{125}I] Triiodothyronine
**Vehicle:**	1% DMSO	**Non-Specific Ligand:**	1.0 µM Triiodothyronine
**Incubation Time/Temp:**	18 hours @ 4°C	**Specific Binding:**	77% *
**Incubation Buffer:**	20 mM Tris-HCl, pH 7.6, 50 mM NaCl, 10% Glycerol, 2 mM EDTA, 5 mM DTT	**Quantitation Method:**	Radioligand Binding
**Kd:**	0.034 nM *	**Significance Criteria:**	≥50% of max stimulation or inhibition
		**Bmax:**	0.16 pmole/mg Protein *

■ 220320 Transporter, Dopamine (DAT)

**Source:**	Human recombinant CHO-K1 cells	**Ligand:**	0.15 nM [^{125}I] RTI-55
**Vehicle:**	1% DMSO	**Non-Specific Ligand:**	10.0 µM Nomifensine
**Incubation Time/Temp:**	3 hours @ 4°C	**Specific Binding:**	90% *
**Incubation Buffer:**	50 mM Tris-HCl, pH 7.4, 100 mM NaCl, 1 µM Leupeptin, 10 µM PMSF	**Quantitation Method:**	Radioligand Binding
**Kd:**	0.58 nM *	**Significance Criteria:**	≥50% of max stimulation or inhibition
		**Bmax:**	0.047 pmole/mg Protein *

* Historical Values

Methods

■ 226400 Transporter, GABA

**Source:**	Wistar Rat cerebral cortex	**Ligand:**	6.0 nM [^{3}H] GABA
**Vehicle:**	1% DMSO	**Non-Specific Ligand:**	10.0 µM NO-711
**Incubation Time/Temp:**	20 minutes @ 25°C	**Specific Binding:**	80% *
**Incubation Buffer:**	10 mM HEPES, pH 7.5, 120 mM NaCl, 4 mM $Ca(CH_3COO)_2$, 10 µM Isoguvacine, 10 µM S(-)-Baclofen	**Quantitation Method:**	Radioligand Binding
**Kd:**	0.30 µM *	**Significance Criteria:**	≥50% of max stimulation or inhibition
		**Bmax:**	60.0 pmole/mg Protein *

■ 204410 Transporter, Norepinephrine (NET)

**Source:**	Human recombinant MDCK cells	**Ligand:**	0.20 nM [^{125}I] RTI-55
**Vehicle:**	1% DMSO	**Non-Specific Ligand:**	10.0 µM Desipramine
**Incubation Time/Temp:**	3 hours @ 4°C	**Specific Binding:**	75% *
**Incubation Buffer:**	50 mM Tris-HCl, pH 7.4, 100 mM NaCl, 1 µM Leupeptin, 10 µM PMSF	**Quantitation Method:**	Radioligand Binding
**Kd:**	0.024 µM *	**Significance Criteria:**	≥50% of max stimulation or inhibition
		**Bmax:**	2.50 pmole/mg Protein *

■ 274030 Transporter, Serotonin (5-Hydroxytryptamine) (SERT)

**Source:**	Human recombinant HEK-293 cells	**Ligand:**	0.40 nM [^{3}H] Paroxetine
**Vehicle:**	1% DMSO	**Non-Specific Ligand:**	10.0 µM Imipramine
**Incubation Time/Temp:**	60 minutes @ 25°C	**Specific Binding:**	95% *
**Incubation Buffer:**	50 mM Tris-HCl, pH 7.4, 120 mM NaCl, 5 mM KCl	**Quantitation Method:**	Radioligand Binding
**Kd:**	0.078 nM *	**Significance Criteria:**	≥50% of max stimulation or inhibition
		**Bmax:**	4.40 pmole/mg Protein *

* Historical Values

Reference Compounds

Cat #	Assay Name	Reference Compound	IC_{50}*	Historical K_i	n_H	Batch *	Concurrent IC_{50}*
200510	Adenosine A_1	R(-)-PIA	0.83 µM	0.49 µM	0.90	314096	0.32 µM
200610	Adenosine A_{2A}	CGS-21680	0.13 µM	0.079 µM	1.0	314151	0.11 µM
200720	Adenosine A_3	IB-MECA	0.78 nM	0.72 nM	0.80	314213	1.36 nM
203100	Adrenergic α_{1A}	Prazosin	0.69 nM	0.28 nM	0.90	314153	0.25 nM
203200	Adrenergic α_{1B}	Prazosin	0.27 nM	0.15 nM	1.0	314154	0.28 nM
203400	Adrenergic α_{1D}	Prazosin	0.88 nM	0.43 nM	0.70	314156	0.75 nM
203620	Adrenergic α_{2A}	Yohimbine	8.40 nM	3.10 nM	0.90	314100	6.13 nM
204010	Adrenergic β_1	S(-)-Propranolol	2.50 nM	1.40 nM	0.80	314161	1.03 nM
204110	Adrenergic β_2	S(-)-Propranolol	0.78 nM	0.54 nM	1.20	314163	0.34 nM
285010	Androgen (Testosterone) AR	Testosterone	6.50 nM	4.30 nM	1.0	314097	2.56 nM
212510	Bradykinin B_1	(Des-Arg^{10})-Kallidin	0.87 nM	0.22 nM	1.10	314221	0.40 nM
212620	Bradykinin B_2	Bradykinin	1.80 nM	1.10 nM	1.0	314223	1.55 nM
214510	Calcium Channel L-Type, Benzothiazepine	Diltiazem	0.036 µM	0.032 µM	0.90	314226	0.021 µM
214600	Calcium Channel L-Type, Dihydropyridine	Nitrendipine	0.72 nM	0.46 nM	0.90	314169	0.25 nM
216000	Calcium Channel N-Type	ω-Conotoxin GVIA	0.034 nM	0.028 nM	1.60	314227	0.022 nM
217030	Cannabinoid CB_1	R(+)-WIN-55,212-2	0.20 µM	0.15 µM	0.70	314171	0.19 µM
219500	Dopamine D_1	R(+)-SCH-23390	1.40 nM	0.70 nM	0.90	314173	1.53 nM
219700	Dopamine D_{2S}	Spiperone	0.25 nM	0.089 nM	1.0	314174	0.26 nM
219800	Dopamine D_3	Spiperone	0.36 nM	0.12 nM	0.90	314297	0.32 nM
219900	Dopamine $D_{4.2}$	Spiperone	0.50 nM	0.20 nM	0.90	314238	0.36 nM
224010	Endothelin ET_A	Endothelin-1	0.23 nM	0.14 nM	1.10	314240	0.10 nM
224110	Endothelin ET_B	Endothelin-1	0.13 nM	0.060 nM	0.90	314241	0.052 nM
225510	Epidermal Growth Factor (EGF)	EGF (human)	1.60 nM	1.10 nM	1.10	314243	3.27 nM
226010	Estrogen ERα	Diethylstilbestrol	0.77 nM	0.22 nM	1.0	314245	0.59 nM
226600	$GABA_A$, Flunitrazepam, Central	Diazepam	0.016 µM	0.013 µM	0.80	314115	0.011 µM
226500	$GABA_A$, Muscimol, Central	GABA	0.032 µM	0.026 µM	0.90	314175	0.032 µM
228610	$GABA_{B1A}$	CGP-54626	6.40 nM	2.90 nM	1.0	314248	4.92 nM
232030	Glucocorticoid	Dexamethasone	3.80 nM	1.80 nM	0.90	314321	4.49 nM
232700	Glutamate, Kainate	L-Glutamic acid	0.24 µM	0.17 µM	0.80	314298	0.21 µM
232810	Glutamate, NMDA, Agonism	L-Glutamic acid	0.41 µM	0.37 µM	0.90	314451	0.18 µM
232910	Glutamate, NMDA, Glycine	MDL 105,519	0.022 µM	0.021 µM	0.60	314253	7.79 nM
233000	Glutamate, NMDA, Phencyclidine	Dizocilpine ((+)-MK-801)	5.10 nM	3.40 nM	0.70	314178	3.66 nM
239610	Histamine H_1	Pyrilamine	3.30 nM	1.60 nM	1.0	314180	2.09 nM
239710	Histamine H_2	Tiotidine	0.022 µM	0.018 µM	1.10	314258	0.013 µM
239820	Histamine H_3	R(-)-α-Methylhistamine (RAMH)	2.30 nM	1.10 nM	1.10	314302	1.69 nM
241000	Imidazoline I_2, Central	Idazoxan	0.012 µM	8.0 nM	1.0	314181	8.80 nM
243520	Interleukin IL-1	IL-1β	0.19 nM	0.14 nM	1.30	314186	0.33 nM

* Batch: Represents compounds tested concurrently in the same assay(s).

Reference Compounds

Cat #	Assay Name	Reference Compound	IC_{50}*	Historical K_i	n_H	Concurrent Batch *	IC_{50}*
250460	Leukotriene, Cysteinyl $CysLT_1$	LTD_4	0.70 nM	0.29 nM	1.0	314262	0.99 nM
251600	Melatonin MT_1	Melatonin	0.21 nM	0.11 nM	0.70	314266	0.49 nM
252610	Muscarinic M_1	4-DAMP	4.50 nM	1.10 nM	1.0	314320	5.51 nM
252710	Muscarinic M_2	4-DAMP	0.055 µM	0.023 µM	1.0	314183	0.028 µM
252810	Muscarinic M_3	4-DAMP	5.10 nM	2.50 nM	1.10	314185	3.50 nM
257010	Neuropeptide Y Y_1	Neuropeptide Y (human, rat)	0.22 nM	0.21 nM	1.10	314271	0.46 nM
257110	Neuropeptide Y Y_2	Neuropeptide Y (13-36) (porcine)	0.21 nM	0.12 nM	0.90	314303	0.54 nM
258590	Nicotinic Acetylcholine	Epibatidine	0.076 nM	0.052 nM	0.90	314188	0.14 nM
258700	Nicotinic Acetylcholine α1, Bungarotoxin	α-Bungarotoxin	1.10 nM	0.72 nM	1.10	314189	1.18 nM
260130	Opiate δ_1 (OP1, DOP)	Naltrindole	0.91 nM	0.16 nM	1.0	314191	1.08 nM
260210	Opiate κ(OP2, KOP)	U-69593	0.016 µM	6.40 nM	0.5	314304	0.012 µM
260410	Opiate µ(OP3, MOP)	DAMGO	0.020 µM	8.10 nM	0.60	314194	0.041 µM
264500	Phorbol Ester	PMA	0.79 nM	0.59 nM	1.0	314195	0.90 nM
265010	Platelet Activating Factor (PAF)	PAF	0.28 nM	0.15 nM	0.90	314197	0.22 nM
265600	Potassium Channel [K_{ATP}]	Glyburide	5.70 nM	0.65 nM	0.80	314201	3.79 nM
265900	Potassium Channel hERG	Astemizole	2.60 nM	2.10 nM	1.10	314202	4.66 nM
268420	Prostanoid EP_4	Prostaglandin E_2 (PGE_2)	1.10 nM	0.45 nM	0.90	314208	0.97 nM
268700	Purinergic P_{2X}	α, β-Methylene ATP	0.082 µM	0.018 µM	1.10	314300	0.029 µM
268810	Purinergic P_{2Y}	ATP	0.018 µM	0.018 µM	0.90	314301	0.017 µM
270000	Rolipram	Rolipram	5.70 nM	2.10 nM	1.0	314203	2.45 nM
271110	Serotonin (5-Hydroxytryptamine) $5\text{-}HT_{1A}$	Metergoline	4.10 nM	2.30 nM	0.90	314275	2.16 nM
271700	Serotonin (5-Hydroxytryptamine) $5\text{-}HT_{2B}$	Ketanserin	0.29 µM	0.18 µM	0.60	314205	0.52 µM
271910	Serotonin (5-Hydroxytryptamine) $5\text{-}HT_3$	MDL 72222	0.011 µM	2.50 nM	0.80	314281	0.020 µM
278110	Sigma σ_1	Haloperidol	0.021 µM	8.80 nM	0.90	314095	7.91 nM
255520	Tachykinin NK_1	L-703,606	3.60 nM	2.60 nM	1.0	314269	2.60 nM
285900	Thyroid Hormone	Triiodothyronine	0.034 nM	0.018 nM	1.0	314309	0.031 nM
220320	Transporter, Dopamine (DAT)	GBR-12909	1.70 nM	1.30 nM	0.90	314094	0.61 nM
226400	Transporter, GABA	NO-711	0.20 µM	0.20 µM	1.10	314299	0.42 µM
204410	Transporter, Norepinephrine (NET)	Desipramine	0.93 nM	0.92 nM	0.60	314086	0.66 nM
274030	Transporter, Serotonin (5-Hydroxytryptamine) (SERT)	Fluoxetine	8.60 nM	1.40 nM	0.90	314284	8.69 nM

* Batch: Represents compounds tested concurrently in the same assay(s).
