# Supplementary material for: Lifting the Mask: Identification of New Small Molecule Inhibitors of Uropathogenic Escherichia coli Group 2 Capsule Biogenesis
Source: PLoS One. 2014 Jul 1;9(7):e96054. doi: 10.1371/journal.pone.0096054 (PMC4077706; doi:10.1371/journal.pone.0096054)
Supplement: File S2 — Experimental procedures for aqueous and LB medium solubility, hepatic microsome stability, PAMPA permeability, and plasma stability. Additional methodological details are provided for these assays. (PDF) [file pone.0096054.s002.pdf]

Experimental procedures for aqueous and LB medium solubility, hepatic microsome stability, PAMPA permeability, and plasma stability

Aqueous and LB Medium Solubility: Solubility analysis was performed using a direct UV kinetic solubility method in a 96-well format. All liquid dispense and transfer steps were performed with the Freedom Evo automated liquid handler (Tecan US). Solubility measurements were performed in buffer solution (PBS (pH 7.4) or LB medium), in duplicate. Samples were incubated at room temperature for a minimum of 18 hrs to achieve equilibrium, then filtered (filter plate, pION Inc, P/N 110322) to remove any precipitate formed. The concentration of the compounds was measured by UV absorbance (250-498 nm) using the Infinite M200 (Tecan US) and compared to the spectra of the precipitation-free reference solutions. Spectroscopically pure 1-Propanol (Sigma P/N 256404) was used as a cosolvent to suppress precipitation in the reference solutions. The solubility of each compound was determined using µSOL Evolution Plus software v3.2 (pION Inc) and is expressed as the concentration (µg/mL) of a solute in a saturated solution.

Assay details:

- Diclofenac Na and Dipyridamole were used as standards. Diclofenac Na is highly soluble. Dipyridamole is poorly to moderately soluble.
- Standards and test compound stocks were made in 100% DMSO
- Assay concentration of standards: 500 µM and test compound: 300 µM
- Cosolvent used in the reference solution to suppress precipitation: 1-Propanol
- Assay DMSO final concentration: 1%

Hepatic Microsome Stability: Metabolic stability was assessed in the presence of human liver microsomes (XenoTech, P/N H0630) and mouse liver microsomes (XenoTech, P/N M1000). All liquid dispense and transfer steps were performed with the Freedom Evo automated liquid handler (Tecan US). NADPH, a required cofactor for CYP450 metabolism, was provided by the NADPH Regenerating System, Solutions A (BD Biosciences, P/N 451220) and B (BD Biosciences, P/N 451200). Compound stock solutions were initially prepared in 100% DMSO and subsequently diluted in acetonitrile for the assay. The pH of the reactions was kept at ~ 7.4 with potassium phosphate buffer (BD Biosciences, P/N 451201). The reactions were started after adding NADPH to the reaction plate containing microsomes and compounds and time 0 min aliquots were promptly collected and mixed with ice cold acetonitrile (spiked with internal standards) to quench the reactions. The remainder of the reaction volume was incubated at 37 °C with shaking. Additional aliquots were collected 60 min after the start of the reaction and promptly quenched with ice cold acetonitrile (spiked with an internal standard). Samples were centrifuged at 3000 rpm for 10 min. The amount of compound in the supernatant was determined by LC/MS/MS (Applied Biosystems, Sciex API4000 Q-Trap) and the percent of parent compound remaining after 60 min was calculated by the following formula:

$$\text{\% parent compound remaining} = \left[\frac{\text{Concentration at 60 min}}{\text{Concentration at 0 min}} \text{ X 100} \right]$$

All reactions were run in triplicate, except negative controls (no NADPH) which were performed as single reactions. Results reported are the mean of each reaction triplicate, normalized to the internal standard, and expressed as a percent compound remaining after the incubation time.

Assay details:

- Human and Mouse Liver Microsomes: 0.5 mg/mL protein concentration
- NADPH Regenerating System: 1.55 mM NADP+, 1.33 mM glucose-6-phosphate,1.33 mM Magnesium chloride, and 0.4 U/mL glucose-6 phosphate dehydrogenase
- Incubation Temperature: 37 °C
- Incubation Time: 60 min
- Standards: Verapamil-HCl and Testosterone, at 20 µM and 50 µM, respectively
- Test compound at 1 µM
- Assay DMSO final concentration: ≤ 0.5%
- Assay ACN final concentration: ≤ 1.2%

Cellular Permeability: Permeability was assessed using the Parallel Artificial Membrane Permeability Assay, PAMPA in a 96-well format. All liquid dispense and transfer steps were performed with the Freedom Evo automated liquid handler (Tecan US). Measurements were performed in an aqueous buffer solution (System Solution, pION Inc, P/N 110151) at pH 5.0, 6.2, and 7.4, in duplicate. A “sandwich” plate (pION Inc, P/N 110212) consisting of a donor bottom plate and an acceptor filter plate was used. The donor wells contained the compounds in 180 µl system solution, and magnetic stir bars. The filter on the bottom of each acceptor well was coated with GIT-0 lipid (pION Inc, P/N 110669) and filled with 200 µl of Acceptor Sink Buffer, pH 7.4 (pION Inc, P/N 110139) containing a surfactant to mimic the function of serum proteins. The permeation time was 30 min and moderate stirring (equivalent to 40 µm Aqueous Boundary Layer thickness) was applied using the Gut-Box™ (pION, Inc, P/N 110205). After the permeation time, the sandwich was disassembled and the amount of compound present in both the donor and acceptor wells was measured by UV absorbance (250-498 nm) using the Infinite M200 (Tecan US) and compared to spectra obtained from reference standards. Mass balance was used to determine the amount of material embedded in the membrane filter. The effective permeability, Pe, was calculated using the software PAMPA Evolution Plus, version 3.2 (pION Inc).

Assay details:

- Verapamil HCl, Metoprolol, and Ranitidine were used as reference standards
- Verapamil HCl is considered highly permeable
- Metoprolol is considered moderately permeable
- Ranitidine is considered poorly permeable
- Permeation time: 30 min
- Moderate stirring (equivalent to 40 µm ABL, aqueous boundary layer, also known as the unstirred water layer)
- Donor buffer pH: 5.0, 6.2 and 7.4
- Double-Sink: pH gradients between donor and acceptor compartments; acceptor buffer contains chemical sink
- Assay DMSO final concentration : 0.5%

Plasma Stability: Stability of the compound in human plasma (BioChemed Services, P/N 752PR-EK3-PMG) was determined. All liquid dispense and transfer steps were performed with the Freedom Evo automated liquid handler (Tecan US). Plasma was allowed to thaw at room temperature prior to preparing the assay solution of plasma:1X PBS (1:1). The assay solution was warmed up at 37 °C prior of adding the compound. Immediately after compounds were added, time 0 min aliquots were promptly collected and mixed with cold acetonitrile (spiked with an internal standard). The remainder of the reaction volume was incubated at 37 °C with shaking. Additional aliquots were collected 180 min after the start of the reaction and promptly quenched with cold acetonitrile (spiked with an internal standard). Samples were centrifuged at 3000 rpm

for 10 min. The amount of compound in the supernatant was determined by LC/MS/MS (Applied Biosystems, Sciex API4000 Q-Trap) and the percent of parent compound remaining after 180 min was calculated by the following formula:

$$\% \text{ parent compound remaining} \left[\frac{\text{Concentration at 180 min}}{\text{Concentration at 0 min}} \quad \text{X } 100 \right]$$

Results reported are the mean of each reaction duplicate, normalized to the internal standard, and expressed as a percent of compound remaining after the incubation time.

Assay details:

- Human Plasma in K3 EDTA
- Procaine and Procainamide were used as standards. Procaine is highly unstable in human plasma, Procainamide is highly stable in human plasma.
- Assay concentrations of standards and test compound: 1 µM
- Incubation Time: 3 hrs
- Reaction pH: 7.4
- Assay DMSO final concentration: 2.5%
